# Matrix stiffness-dependent STEAP3 coordinated with PD-L2 identify tumor responding to sorafenib treatment in hepatocellular carcinoma

**DOI:** 10.1186/s12935-022-02634-7

**Published:** 2022-10-13

**Authors:** Shunxi Wang, Long Chen, Wanqian Liu

**Affiliations:** 1 Key Laboratory of Biorheological Science and Technology, Ministry of Education, Bioengineering College, Chongqing University, Chongqing, 400044 China; 2 Bioengineering Institute of Chongqing University, 174 Shazheng Street, Chongqing, 400000 China

**Keywords:** Matrix stiffness, Ferroptosis, Tumor immunity, Hepatocellular carcinoma, Cirrhosis

## Abstract

**Background:**

Ferroptosis have been implicated in tumorigenesis, tumor progression, and chemo- and immuno-therapy in cirrhotic hepatocellular carcinoma (HCC), indicating its association with matrix stiffness and clinical benefit of targeting drugs or immune checkpoint inhibitor. Here, we postulated that increased matrix stiffness reduces ferroptosis and impairs tumor immunity by regulating the expression of ferroptosis- and immune-related genes in HCC, which might be a robust predictor of therapeutic efficacy.

**Methods:**

Using publicly available tissue microarray datasets, liver cancer rat model, and clinical specimen, ferroptosis-related differential genes in HCV-infected cirrhotic HCC and its mechanical heterogeneous pattern of expression were screened and identified. Further investigation on the underlying mechanism of matrix stiffness-regulated ferroptosis and the expression of immune mediator were performed. Finally, threshold analysis of HCC cases with sorafenib treatment revealed the value of clinical applications of these potential predictors.

**Results:**

STEAP3 was identified as the ferroptosis-related differential genes in HCV-infected cirrhotic HCC. Stiffer matrix decreased STEAP3 in the invasive front area of HCC and the liver cirrhotic tissue. Contrarily, softer matrix induced STEAP3 in the central area of HCC and the normal liver tissue. Immunological correlation of STEAP3 in cirrhotic HCC showed that STEAP3-mediated immune infiltration of CD4+ T and CD8+ T cells, macrophages, neutrophils, and dendritic cells and HCC prognosis, predicting to regulate immune infiltration. Overexpression of STEAP3 induced ferroptosis and inhibited the expression of immune mediator of PD-L2 on a stiff matrix. Especially, the ferroptosis- and immune-related gene predictive biomarker (FIGPB), including STEAP3 and PD-L2, predicts better clinical benefit of sorafenib in HCC patients.

**Conclusions:**

This finding identifies matrix stiffness impairs ferroptosis and anti-tumor immunity by mediating STEAP3 and PD-L2. More importantly, coordinated with PD-L2, matrix stiffness-dependent STEAP3 could be applied as the independent predictors to favorable sorafenib response, and thus targeting it could be a potential diagnosis and treatment strategy for HCC.

**Supplementary Information:**

The online version contains supplementary material available at 10.1186/s12935-022-02634-7.

## Introduction

Hepatocellular carcinoma (HCC) is the fifth commonest cancer worldwide, and causes nearly 600,000 deaths each year. Approximately 80% of HCC frequently emerge on the background of liver cirrhosis by hepatitis B virus (HBV) or hepatitis C virus (HCV) infection [1]. Early detection and timely intervention are the best strategies to reduce morbidity in HCC patients [2]. Current multiple diagnostic methods including ultrasound, computed tomography, magnetic resonance imaging, selective hepatic angiography, and tumor biomarkers such as alpha-fetoprotein, and therapeutic methods including chemotherapy, immunotherapy, and surgery are widely used in HCC patients [3, 4]. However, HCC still has a poor prognosis and low 5-year overall survival rate, due to delayed diagnosis and limited clinical efficacy. Therefore, the clinical development of diagnostic, prognostic and predictive biomarkers is imminent. Especially, the biomarker for screening the chemotherapy- and immunotherapy-fitted patients remains exploration to guide HCC precision treatment and improve clinical benefit.

Ferroptosis driven by accumulated iron-dependent lipid ROS is a novel form of nonapoptotic regulated cell death, which was first observed in Ras mutant tumor cells by treatment of oncogenic Ras-selective lethal small molecules, such as erastin and RSL3 [5]. Ferroptosis is mainly mediated by mevalonate (MVA) pathway, sulphur-transfer pathway, and the heat-shock factors (HSFs)-HSPB1 pathway in mammalian cells [6]. Among them, the inactivation of STEAP3 (six-transmembrane epithelial antigen of prostate 3) inhibits ferroptosis by blocking the transport of iron [7]. Recent evidences showed that ferroptosis are associated with the pathological process of various diseases, including cirrhosis and HCC [8, 9]. For example, ferritinophagy-mediated ferroptosis of hepatic stellate cells mitigates carbon tetrachloride-induced hepatic fibrosis and reduces the development of HCC [10]. Moreover, sorafenib-induced ferroptosis significantly prolong the survival of advanced HCC patients [11], suggesting that ferroptosis induction may provide a promising new approach to the efficient triggering of cancer cell death. Altogether, a reasonable drug target and predictive biomarkers based on ferroptosis are required to select responsive HCC patients.

Tissue mechanics provide higher-order guidance for development and homeostasis, and are consistently modified in tumor progression. Excessive extracellular matrix (ECM) deposition and cross-linking lead to increased tissue stiffness in various cancer, such as HCC, breast cancers and other solid tumors [12]. Previous reports demonstrated that cells recognize and respond to matrix stiffness by exerting contractile force and sensing counter-tension [13]. Increased matrix stiffness promotes invasion, metastasis and chemoresistance capacity of tumor cells by triggering epithelial-mesenchymal transition (EMT) and activating the related signaling pathway, such as, Wnt/beta-catenin, TGF-beta [14–16]. Moreover, stiffer matrix regulates tumor immunity by activating immune infiltration of myeloid cells and the polarization of macrophage and neutrophil [17, 18]. Altogether, growing evidences have shown that matrix stiffness controls the tumorigenesis, progression and occurrence of HCC. However, the effect of matrix stiffness on ferroptosis and its immunity response in cirrhotic HCC remains unclear.

In addition, the tumor microenvironment (TME) contains a variety of cells (e.g., immune cells, stromal cells, endothelial cells, and cancer-associated fibroblasts), which facilitate tumor progression. Among them, infiltrating immune cell interactions with other components by immunoactivators and immunosuppressors in TME can impact tumor fate [19]. New therapeutic strategies target immune cells within the TME, including checkpoint blockade and chimeric antigen receptor (CAR) T cell therapies, have proven to be a promising treatment for various cancers. Antibodies targeting other immune checkpoints, such as programmed cell death-1 (PD-1) and its ligand (PD-L1), now have multiple approvals in patients with NSCLC, CRC, and other cancers [20, 21]. Recent studies reported that immune-related prognostic biomarker (e.g., PD-L1, PD-L2, SFRP4, CPXM1, and COL5A1) might bring some potential implications in the precision treatment of various cancers [22–24]. Therefore, exploring tumor immune microenvironment associated with tumor tissue mechanics and ferroptosis contributes to improved outcomes in patients with advanced HCC.

Given CD8^+^ T cells induced ferroptosis to suppress tumor growth by IFN-γ, the effect of immunomediator on ferroptosis and its predictive value emergently need to be explored [25]. Here, we investigated whether matrix stiffness mediates ferroptosis- and immune-related genes expression in cirrhotic HCC, which participates in the regulation of ferroptosis and tumor immune environment, and affects patient prognosis and sorafenib efficacy. We first screened out differentially ferroptosis- and immune-related genes STEAP3, and PD-L2, and verified mechanical heterogeneity of these gene expressions in HCC. Next, the effect of matrix stiffness on ferroptosis and the expression of PD-L2, and the underlying molecular mechanism were determined in vitro. We further explored the clinical value of matrix stiffness-dependent ferroptosis- and immune-related gene STEAP3 and PD-L2 as predictor for prognosis and clinical benefit of sorafenib treatment in cirrhotic HCC patients. Collectively, these findings demonstrated that matrix mechanics mediated the ferroptosis of HCC cells and tumor immune environments through STEAP3 and PD-L2. Our study identifies that matrix stiffness-dependent STEAP3 and PD-L2 could be applied as the independent predictors to sorafenib response, and thus targeting it could be a potential diagnosis and treatment strategy for HCC.

## Materials and methods

### Patients

In March 2021, HCC tissues were obtained from the Department of Hepatobiliary Surgery, Chongqing University Three Gorges Hospital (CUTGH), and collected during surgery and fixed in 4% paraformaldehyde. Clinical data were available to obtain from hospital records. This research was supported by the Independent Ethics Committee (IEC) of the CUTGH and all patients were well informed of storing and upcoming use of their resected specimens for further research purposes.

### Sequencing datasets

Three independent microarray data (accession number: GSE45050, GSE17548 and GSE109211) were downloaded from the Gene Expression Omnibus (GEO) database (https://www.ncbi.nlm.nih.gov/geo/), and the quality of the data was assessed by RMAExpress software (http://rmaexpress.bmbolstad.com). Quality evolution of the Affymetrix datasets was carried out by affyPLM (http://www.bioconductor.org). Class comparison analysis were performed by BRB-Array tools (http://linus.nci.nih.gov/BRB-ArrayTools.html; Version 4.2.0) after normalization with RMAExpress software. RNA sequencing and clinical data of 160 normal people and 369 HCC patients were obtained from the TCGA data portal (http://cancergenome.nih.gov/). The GSE45050 dataset included 3 normal samples, 5 liver cirrhotic samples and 6 HCC tissue samples. Expression data from the GSE17548 dataset included 3 cirrhosis samples with HCV positive and 3 HCV-infected HCC samples. The GSE109211 dataset included 21 responders and 46 non-responders in hepatocellular carcinoma with sorafenib treatment in the phase 3 STORM trial.

### Identification of differential expression genes and clinical prognosis analysis

The identification of differentially expressed genes (DEGs) in normal control (NC), liver cirrhotic (LC) and HCC tissues from two independent microarray data (accession number: GSE45050, GSE17548) were detected by the empirical Bayes method [26]. A false discovery rate (FDR) adjusted *P-valu*e < 0.01 and |log fold change| ≥ 1.0 was set as the threshold for differentially expressed genes. Meanwhile, the expression analysis of DEGs was performed by using the free online web platform at http://gepia.cancer-pku.cn/index.html. Briefly, the expression of each indicated gene was evaluated in LIHC, data set matched with ‘TCGA normal and GTEx data’ set (normal tissue), with a *|Log*_*2*_*FC| Cutoff* of 1, a *p-value Cutoff* of 0.01, and results showed using a *log2 (TPM* + *1)* log scale. The overall survival curves were generated by using the following parameters: Group Cutoff = Median, with Cutoff-High (%) = 50 and Cutoff-Low (%) = 50; Hazards Ratio (HR) = Yes.

### STEAP3 co-expression network selection and gene function enrichment analysis

The genes co-expressed with STEAP3 in HCC were screened according to the Pearson correlation coefficient (p < 0.05) and plotted in the volcano plot by the “ggplot2” package. Heatmaps of the top 50 negative or the top 50 positive STEAP3 expression correlated genes were plotted by the “pheatmap” R package. LinkedOmics was employed to gain insights into the biological functions of those co-expressed genes of STEAP3.

### Correlation analysis of STEAP3 with immune cells infiltration

The abundance of immune infiltrations was estimated by TIMER, which is a comprehensive resource for systematic analysis of clinical impact across diverse cancer types (https://cistrome.shinyapps.io/timer/), including 10,897 samples across 32 cancer types from The Cancer Genome Atlas (TCGA). Simply, the abundance of six immune cell types: B cell, CD4^+^ T cell, CD8^+^ T cell, neutrophil, macrophage and dendritic cell in the tumor microenvironment is estimated. The correlations between STEAP3 and infiltrating levels of various immune cells in HCC were checked. The expression level (displayed with log_2_ RSEM) and copy number of STEAP3 was shown on X axis, and the infiltration level is shown on Y-axis. An integrated repository portal for tumor-immune system interactions (TISIDB, http://cis.hku.hk/TISIDB/index.php) was utilized to examine tumor and immune system interactions in 28 types of tumor infiltrating lymphocytes (TILs) across human cancers. Then, the correlations between prognostic genes and TILs were measured by Spearman’s test. Some other databases used are listed in Additional file 1: Table S1.

### Animal models

All animal experiments were approved by the Institutional Animal Care and Use Committees and performed in accordance with the Association for Assessment and Accreditation of Laboratory Animal Care guidelines (http://www.aaalac.org). 5-week-old male Sprague Dawley (SD) rats were given a single injection of diethylnitrosamine (DEN) at a dose of 100 mg/kg body weight as an initiation of liver carcinogenesis. Rats were administered 40 ppm *N*-nitrosomorpholine (NMOR) in drinking water for 14, 20 and 24 weeks. An interim sacrifice was performed at week 14, 20 and 24. After liver tumors were excised, plane sections of the livers were prepared for each rat, processed for production of paraffin sections, and stained with hematoxylin and eosin (HE).

### Cell culture

HepG2 cell lines (HB-8065) was purchased from the American Type Culture Collection (ATCC). The malignant human hepatoma cell line HCCLM3 was obtained from Nanjing Kebai Biotechnology Co., Ltd (CBP60654). HepG2 and HCCLM3 cells lines were cultured in high-glucose DMEM (Gibco) supplemented with 10% fetal bovine serum (FBS, Gibco), 100 U/mL penicillin, and 100 μg/mL streptomycin.

### Preparation of matrix with different stiffness

Matrix with various stiffness was prepared by adjusting the chemical composition of polyvinylalcohol (PVA) solution according to Additional file 1: Table S1 [27]. Briefly, 8 g PVA powder (Aladdin, Los Angeles, CA, USA) was dissolved in 100 mL water and stirred for 2 h at 95 °C to obtain PVA solution. 37% HCl (catalyst) and 25% Glutaraldehyde (Ourchem, Shanghai, China) with the desired composition (Additional file 1: Table S2) were mixed to 8% PVA hydrogel of 10 mL. The hydrogels were soaked in 75% ethanol and then incubated with *N*′-a-hydroxythylpiperazine-*N*′-ethanesulfanic acid (HEPES, pH 7.2–7.4, H3375, Sigma-Aldrich, St. Louis, MO, USA) for 45 min, and were washed by sterilized HEPES three times. Next, Collagen-I (10 μg/mL, Corning, China) of rat tail was cross-linked to the PVA gel surface at 4 °C for 12 h by the addition of Sulfo-SANPAH (1 mg/mL in HEPES, Thermo, USA) to facilitate the cell adhesion. Finally, the gels were washed twice with phosphate buffer solution (PBS) to remove unreacted fibronectin before cell culture.

### Quantitative PCR analysis

Total cellular RNA was extracted from cells with the RNeasy Mini Kit (TIANGEN, DP419, China) according to the manufacturer's guidelines. Then, 1 μg of total RNA was reversed transcript to cDNA using the RevertAid RT Reverse Transcription Kit (Thermo Scientific, Waltham, MA). Quantitative PCR (qPCR) was performed using iQSYBR Green Master Mix (Bio-rad) on the CFX96 Touch Quantitative PCR Detection System. Gene expression was quantified using the primers from Additional file 1: Table S3.

### Western blotting

Cells were dissolved in RIPA buffer (10 mM Tris–HCl pH 7.5, 1% sodium deoxycholate, 150 mM NaCl, 0.1% SDS, 1% Triton X-100, and 5 mM EDTA) plus a protease inhibitor). Protein concentrations were quantified using a BCA Protein Assay Kit (Beyotime Biotechnology). The same amount of protein (40 μg) was separated by SDS-polyacrylamide gel electrophoresis and subsequently transferred to a polyvinylindene fluoride (PVDF) membranes (Immobilon-P^SQ^). After transfer, the membranes were blocked with 5% skim milk powder in TBST buffer (50 mM Tris–HCl, 100 mM NaCl, and 0.1% Tween-20, pH7.4) at RT for 1 h and were incubated with the following antibodies at the indicated concentrations overnight at 4 °C. The antibodies included anti-GAPDH (Gene ID: 2597, ZEN-BIO); anti-STEAP3 (Cat No: 17186-1-AP, Proteintech); anti-SLC3A2 (Cat No: 66883-1-lg, Proteintech); anti-GPX4 (Cat No: 67763-1-lg, Proteintech); anti-Ferritin (Gene ID: 2512, Beyotime); anti-PDL2 (Gene ID: 58205, ThermoFisher). Proteins were visualized using ECL plus fluorescence detection reagent (Thermo Scientific, Waltham, MA).

### Immunohistochemical staining

IHC staining was performed on representative tissue sections from formalin-fixed (4%) and paraffin-embedded tissue blocks from mice hepatic tumor tissue. Simply, sections were first deparaffinized in xylene, hydrated in gradient alcohol followed by antigen retrieval in citric acid buffer (pH 6.0). After washing with PBS (3 × 5 min), sections were incubated with 3% H_2_O_2_ for 10 min and then blocked with blocking reagent (QuickBlock™ Blocking Buffer, Beyotime, Beijing, China) for 30 min at 37 °C. Thereafter, the sections were covered overnight at 4 °C using the following antibodies at the indicated concentrations: anti-STEAP3 (Proteintech) 1:20; anti-GPX4 (Proteintech) 1:50. Relevant second antibody IgG HRP (Horse-Radish Peroxidase) were incubated for 30 min at 37 °C. Subsequently, samples were developed by incubation in diaminobenzidine (DAB) Detection Kit (PV9000, ZSGB-BIO, China). The stained slides were mounted for observation and all images were taken using bright-field microscopy (Leica, Germany).

### Immunofluorescence staining

Liver cancer cells with different matrix stiffness were fixed in 4% (v/v) paraformaldehyde (Solarbio, Beijing) solution at room temperature for 15 min, permeabilized with 0.2% (v/v) Triton X-100 (Solarbio, Tongzhou, BJ) for 15 min, washed with PBS three times, and blocked with 2% (w/v) BSA (Solarbio, Beijing) at 37 °C for 30 min. Then, cells were incubated with primary antibodies against SLC7A11 (Proteintech, 1:100), STEAP3 (Proteintech, 1:100), PD-L1 (Proteintech, 1:100), PD-L2 (Thermo, 1:100) and at 4 °C overnight, followed by an incubation with goat anti-rabbit IgG (H&L) Alexa Fluor 488 secondary antibodies (Beyotime, Jiangsu) and donkey anti-rabbit Alexa Fluor 555 secondary antibodies (Beyotime, Jiangsu) at 37 °C for 30 min. Nuclei were stained with DAPI (Cell Signaling, Danvers, MA). Images were obtained by using a laser confocal fluorescent microscope (Leica, Germany).

### Young’s modulus analysis

The Young’s modulus of tumor center and invasive frontier area of HCC tissue was measured by atomic force microscopy (AFM, JPK Instruments, NanoWizard II, Germany). The silicon nitride spherical probe used in the experiment was purchased from Nanosensors (Neuchatel, Switzerland) with a diameter of 4 µm, a spring constant of 0.2 N/m, and a Poisson’s ratio of 0.5. The selected tapping mode was used to detect Young’s modulus. Finally, the Young’s modulus of liver tumor tissue was calculated by Force–displacement curves.

### Statistical analysis

Statistical analysis was performed by GraphPad Prism (version 8, San Diego California USA). All experiments were analyzed with a minimum of three independent repeats. Statistical tests applied were One-Way analysis of variance (ANOVA) by Tukey-test. Data are presented as mean ± standard error of mean (SEM). *P < 0.05, **p < 0.01, ***p < 0.001, ****p < 0.0001.

## Results

### Differential expression pattern of STEAP3 in HCV-infected cirrhotic HCC

**Fig. 1:**
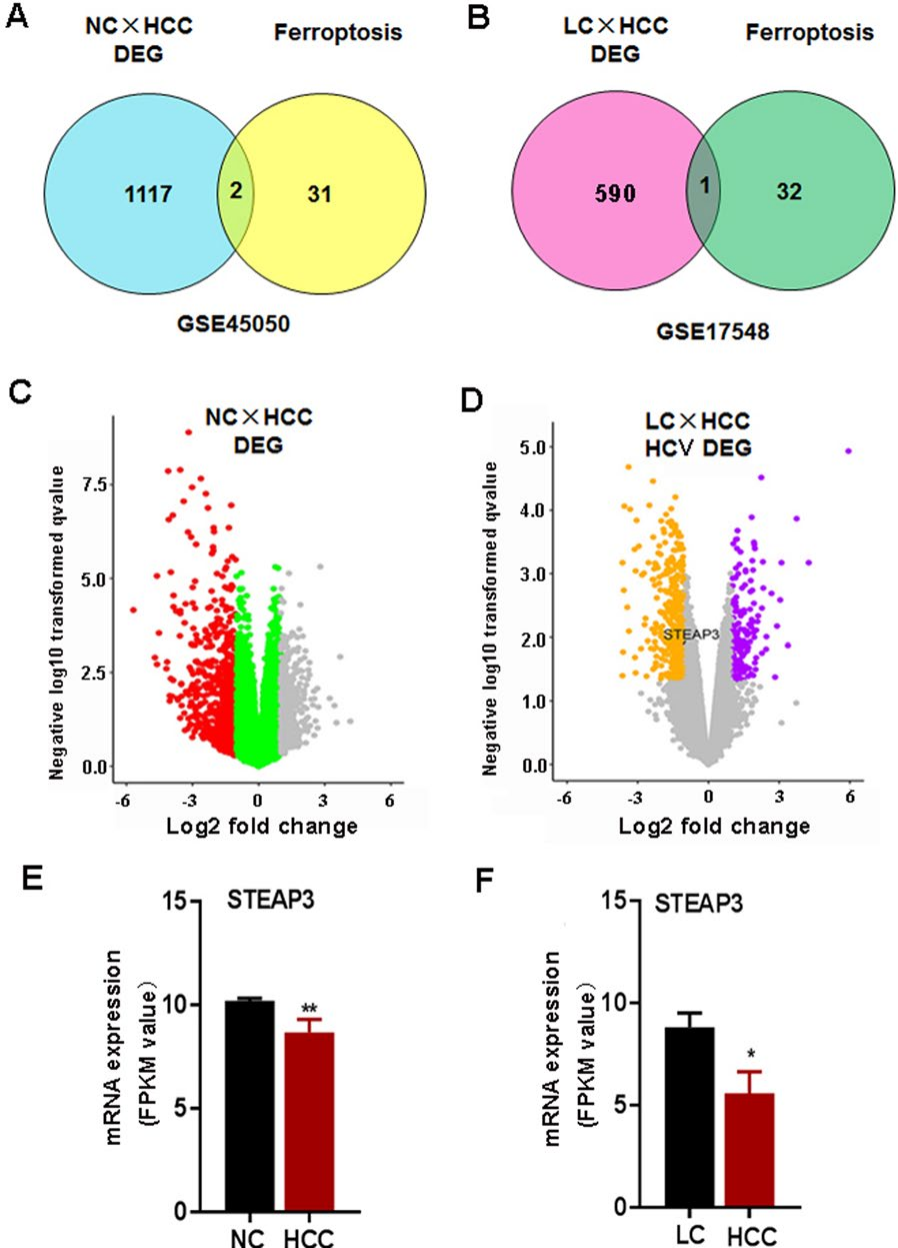
Expression profile of ferroptosis-related DEGs in normal liver tissue vs. HCC and HCV- induced cirrhosis vs. HCC from GEO datasets. **A**, **B** The venn diagrams of the expression of differential gene STEAP3 in normal liver tissue, cirrhosis and HCC tissues. **C**, **D**. The volcano diagrams of the expression of differential gene STEAP3 in normal liver tissue, cirrhosis and HCC tissues. **E** The RT-PCR results showing the expression level of STEAP3 in normal liver tissues and HCC tissues. **F** The RT-PCR results showing the expression level of STEAP3 in HCV-induced cirrhosis and HCC tissues

To identify the expression pattern of ferroptosis-related genes in HCC, mRNA datasets (GSE45050) and 33 ferroptosis-related genes were screened. The screening results showed that there were 1119 DEGs and 2 ferroptosis-related DEGs (SLC7A11 and STEAP3) in HCC vs. normal liver tissue (Fig. 1A, C and Additional file 2: Fig. S1A). To validate the expression difference of SLC7A11 and STEAP3 in HCC, the mRNA level was analyzed. Compared with the normal liver tissues, SLC7A11 was significantly upregulated and STEAP3 was downregulated in HCC (GSE45050; Fig. 1E and Additional file 1: Table S4). Compared with the liver cirrhosis tissues, SLC7A11 was also significantly upregulated and STEAP3 was downregulated in HCC (GSE45050; Additional file 1: Table S5). To further confirm these DEGs in cirrhotic HCC caused by hepatitis C, mRNA datasets (GSE17548) and 33 ferroptosis-related genes were screened. The results showed that there were 590 DEGs and 1 ferroptosis-related DEGs (STEAP3) in HCV-infected cirrhotic HCC (Fig. 1B, D and Additional file 2: Fig. S1B). To validate the expression difference of STEAP3 in cirrhotic HCC, the mRNA level was analyzed. The mRNA expression of STEAP3 was significantly downregulated in HCV-infected cirrhotic HCC compared with the cirrhosis tissues, and SLC7A11 was no significant in HCV-infected cirrhotic HCC (Fig. 1F and Additional file 1: Table S6). These results suggested that there was a significant difference in the expression of ferroptosis-related genes STEAP3 in HCV-infected cirrhotic HCC.

### Prognostic significance of STEAP3 in HCC patients

**Fig. 2:**
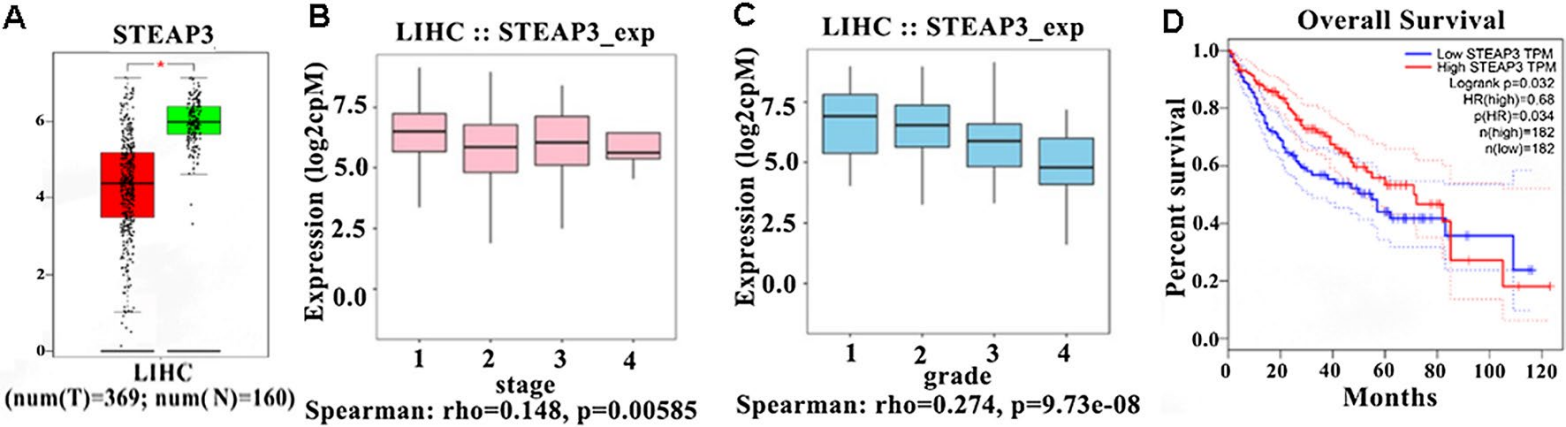
Prognostic significance of STEAP3 in HCC patients. **A** The box plots showing the gene expression of STEAP3 in HCC (369 samples) vs. normal liver tissues (160 samples). **B**, **C** Boxplots for STEAP3 gene expression in different tumor stages (TS) and tumor grades (TG) in HCC patients. **D** Analysis of STEAP3 gene expression with survival prognosis in HCC patients

To investigate the prognostic significance of STEAP3 in HCC, gene expression profile, mutation spectrum, and methylation were comprehensively analyzed (Additional file 3: Fig. S2). The heatmap showed that the expression of ferroptosis-related genes was mainly affected by copy number (Additional file 3: Fig. S2B). Compared with the normal tissues (n = 160), the expression of STEAP3 was downregulated in HCC tissues (n = 369) (Fig. 2A). An approximately downward trend with STEAP3 in different tumor stages (TS) and tumor grades (TG) of HCC patients were observed, respectively (Fig. 2B, C). To further evaluate the role of STEAP3 expression on the prognosis of HCC patients, mRNA data and overall survival (OS) time from TCGA databases were evaluated. Survival analysis showed that HCC patients with low expression of STEAP3 had a short OS time (Fig. 2D). These results suggested that STEAP3 was the potential biomarker of the prognosis in HCC patients.

Given mutation implicated in the survival and recurrence of cancer patients [28], the mutation landscape of 33 ferroptosis-related genes in HCC samples was analyzed. It was found that the mutation rates of STEAP3 in HCC was 6% (Additional file 3: Fig. S2A). To explore the effect of STEAP3 mutation on mRNA expression and DNA methylation, cBioPortal databases were used. The results showed that the mRNA expression was significantly up-regulated (P < 0.0001) after STEAP3 mutation, but the methylation level was significantly down-regulated in HCC (P < 0.0001) (Additional file 4: Fig. S3A, B). The altered STEAP3 was no significant effect (P = 0.431) on the survival and prognosis of patients (Additional file 4: Fig. S3C).

Given DNA methylation, most frequently occurred at CpG dinucleotides, correlated to mRNA expression and prognosis of cancer patients [29], methylation analysis with Mexpress databases was performed. The methylation heatmap showed that the expression of ferroptosis-related genes was affected by methylation, and its methylation degree was significantly varied (Additional file 3: Fig. S2C). Accordingly, a total of 3 types’ methylations of STEAP3 were obtained. Among them, cg26888222 predicted a worse prognosis (Additional file 4: Fig. S3D). To further predict the sensitive and specific of survival, the area under the curves (AUC) was assessed by receiver operating characteristic (ROC) curve analysis. The results showed that the AUC of STEAP3 was 0.846 (Additional file 4: Fig. S3E). All the above, these results suggested that STEAP3 was associated with overall survival of HCC patients.

### STEAP3 co-expression network in hepatocellular carcinoma

**Fig. 3:**
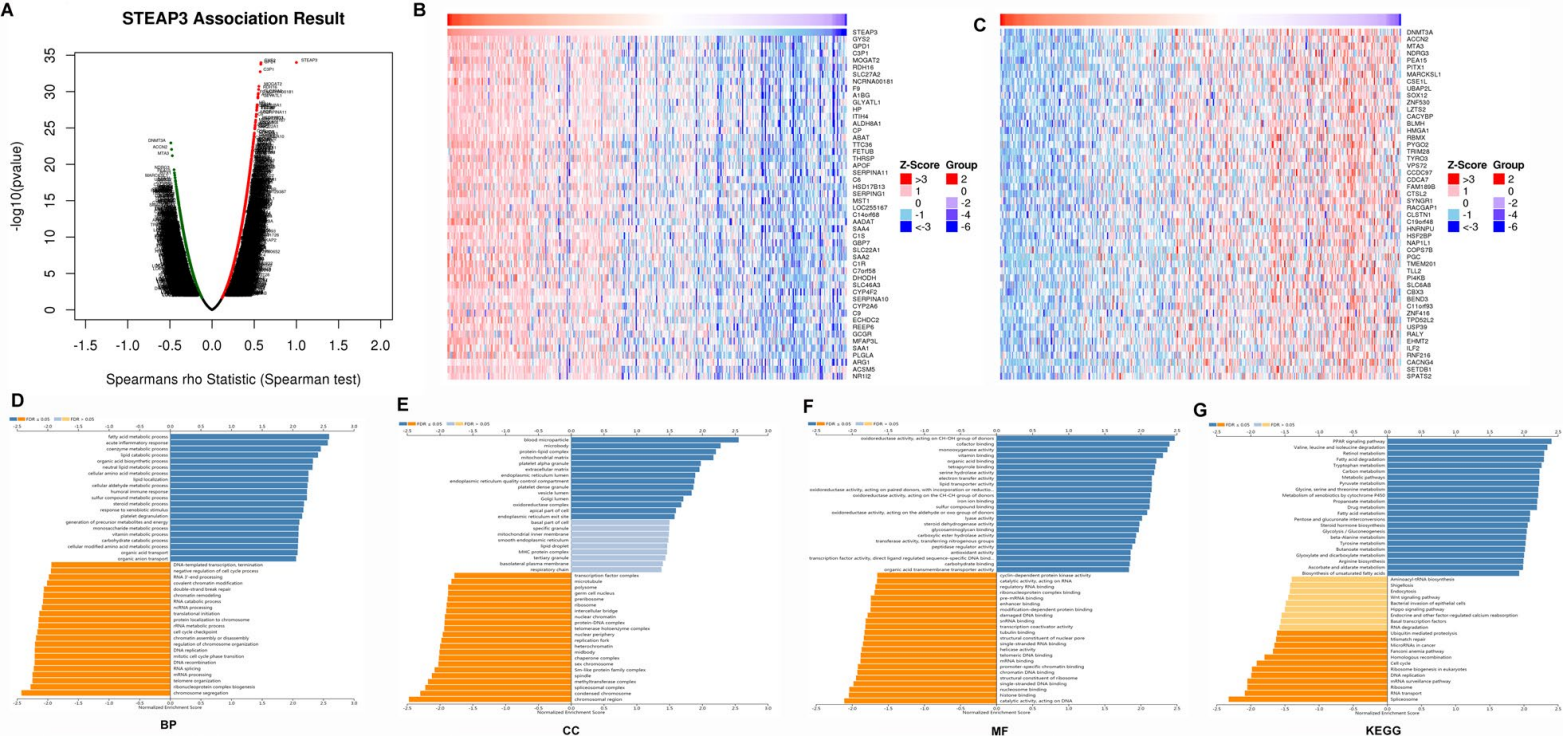
Co-expression genes of STEAP3 in HCC. **A** The whole significantly correlated genes with STEAP3 identified with LinkedOmics in LIHC. **B**, **C** The top 50 genes displaying positive and negative association with STEAP3 in LIHC were showed in heatmaps respectively. The red represented positively connected genes and the blue meant negatively connected genes, respectively. **D**–**G** GO annotations as well as KEGG pathways of STEAP3 in LIHC cohort

For investigating the biological meaning of STEAP3 in LIHC progression, the STEAP3 co-expression pattern in the TCGA-LIHC cohort was checked by deploying the function module of LinkedOmics. As shown in Fig. 3A, it showed that 3928 genes (red dots) positively related with STEAP3, and 5836 genes (green dots) negatively associated (p < 0.05), respectively. The top 50 genes bearing positive and negative association with STEAP3 were shown in the heatmaps (Fig. 3B, C). Gene Ontology term annotation showed that STEAP3-coexpressed genes joined mainly in lipid catabolic process, collagen-containing extracellular matrix, iron ion binding, lipid transporter activity (Fig. 3D–G), indicating the expression of STEAP3 are association with ferroptosis and matrix stiffness.

### Tunable mechanical properties of the PVA hydrogel

**Fig. 4:**
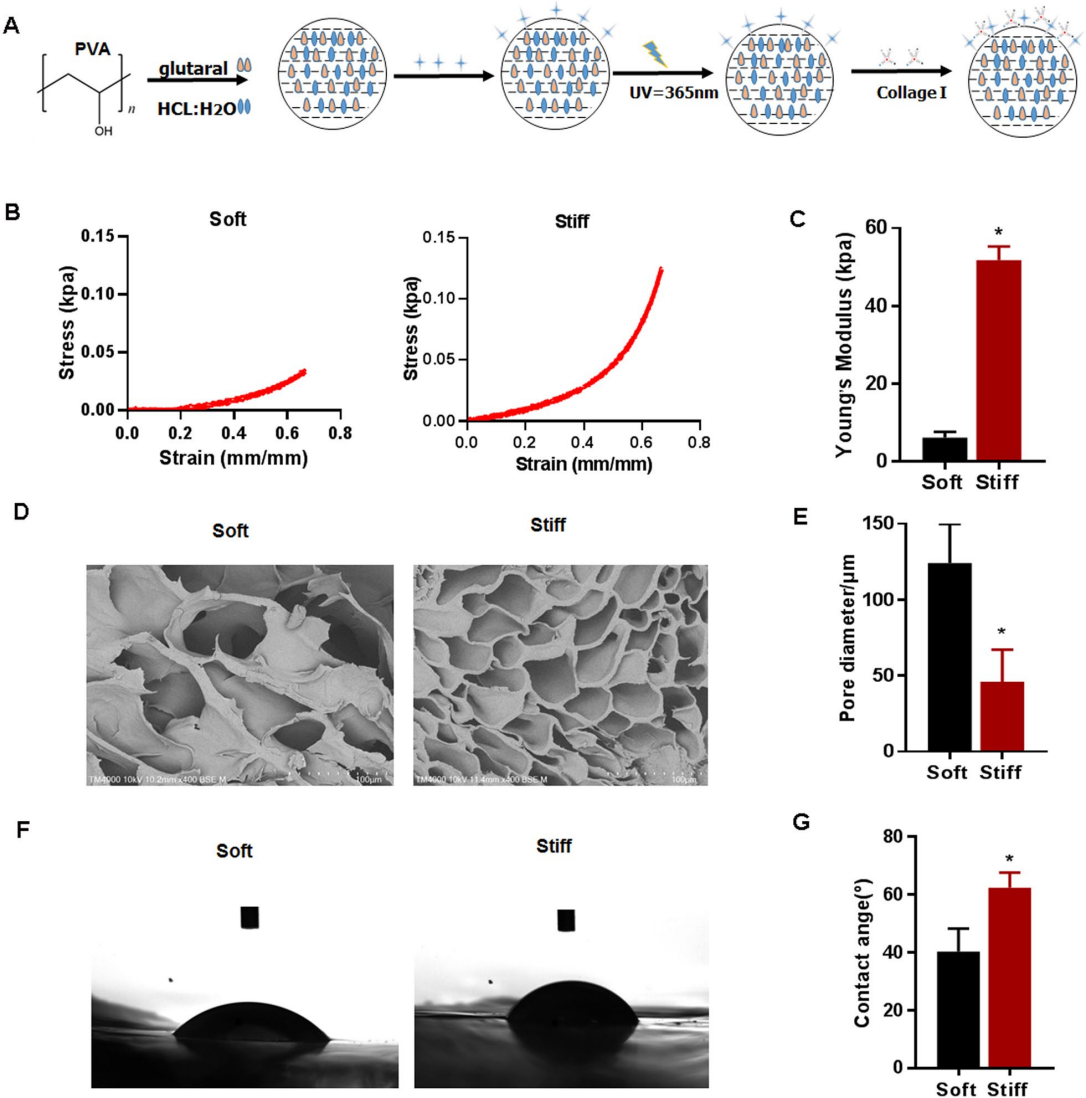
Characterization of mechanical tunable PVA hydrogels. **A** A schematic of PVA hydrogel crosslinking and coating. **B**, **C** The young modulus of PVA hydrogels contain different concentration of crosslinkers. **D**, **E** Pore size of dried soft and stiff hydrogel substrates statisticed by SEM images. **F**, **G** The contact angle of swollen soft and stiff hydrogels substrates statisticed

To explore the effect of matrix stiffness on the ferroptosis of HCC cells, mechanically tunable PVA hydrogels were used in this study. The process for producing the PVA hydrogels is shown in Fig. 4A. Two hydrogels with different stiffnesses were used to simulate the liver at different stages (2 kPa for a normal liver, and 40 kPa for cirrhotic HCC). Given the same concentration of the PVA solution (8 wt%), the young modulus showed a highly significant increase when the stiff group was compared to the soft group (Fig. 4B, C). With the increase of the young modulus, the structure of hydrogel changed. The pore size of the dried hydrogels significantly increased when the soft samples was compared to the stiff group (Fig. 4D, E). The contact angle of hydrogels significantly increased when the stiff group was compared to the soft group (Fig. 4F, G). The PVA gels used in this study were coated with collagen I, which is the predominant ECM protein in the liver. Cells of the HCC cell line HCCLM3 and HepG2 were then seeded on matrices with different stiffnesses.

### Mechanical heterogeneity of STEAP3 expression in cirrhotic HCC

**Fig. 5:**
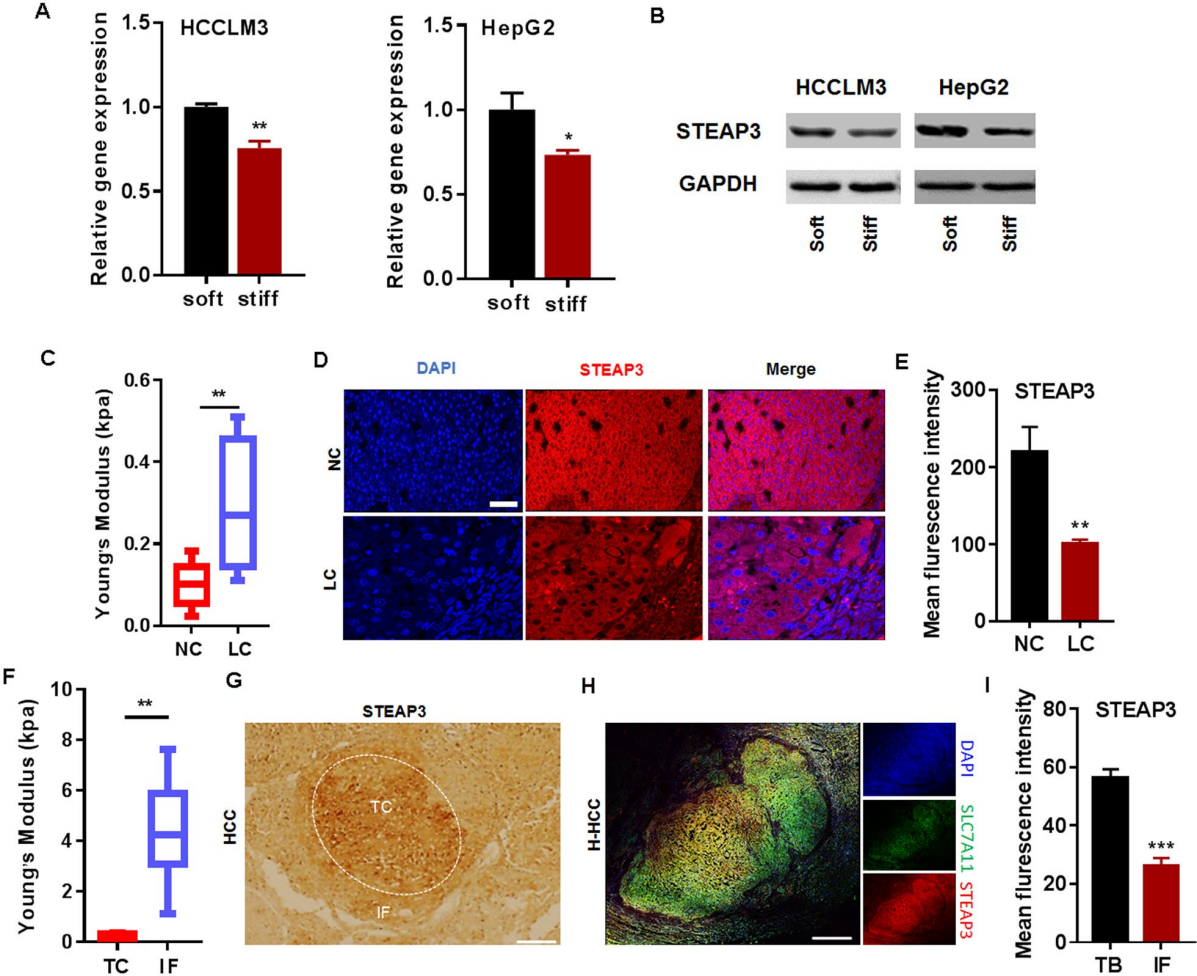
Matrix stiffness mediates the expression of STEAP3 in cirrhotic HCC. **A**, **B** The effects of matrix stiffness on mRNA and protein expression of STEAP3 in HCCLM3 and HepG2 cells cultured on PVA gels (2 and 40 kPa). **C** Analytic results of AFM showing young modulus of cirrhotic tissue and normal liver tissue. **D** Representative immunofluorescence images showing the expression of STEAP3 in normal liver tissue and cirrhosis tissue of SD rat. Bar: 25 μM. **E** Fluorescence statistical results showing the expression level of STEAP3 in normal liver tissue and cirrhosis tissue. **F** Analytic results of AFM showing young modulus of the IF and TC area in HCC tissues of SD rat. **G** Representative immunohistochemical staining images showing the expression of STEAP3 in TC and IF region from HCC tissue of SD rat. Bar: 500 μm. **H** Representative immunofluorescence images of STEAP3 in TC and IF region of cirrhotic HCC specimen. Bar: 500 μm. **I** Fluorescence statistical results showing the expression level of STEAP3 in IF and TC region. All data were presented as mean ± SEM, *N* ≥ 3. *p < 0.05; **p < 0.01; ***p < 0.001

To verify whether the expression of STEAP3 is mediated by matrix stiffness, two human HCC cell lines, HCCLM3 and HepG2, were cultured and checked on tunable PVA hydrogel with different stiffness (Additional file 1: Table S2). The mRNA and protein level of STEAP3 on stiff matrix gels (40 kPa) were significantly lower than those on soft (2 kPa) (Fig. 5A, B).

To further investigate whether matrix stiffness involves in the regulation of STEAP3, we firstly observed its expression in a DEN + NMOR-induced rat model of cirrhosis and HCC. HE staining and masson images showed the lesions of cirrhosis and HCC, especially (Additional file 5: Fig. S4A). AFM results showed that there was a higher young modulus in liver cirrhotic (LC) tissues than in the normal liver tissues (Fig. 5C). Accompanied with the normal liver tissues, STEAP3 expression was significantly decreased in cirrhotic tissues (Fig. 5D, E). Correspondingly, increased young’s modulus from TC to IF region in rat HCC tissues was observed (Fig. 5F). Immunohistochemically staining images showed that STEAP3 expression was decreased in the IF region, compared with the TC region of rat HCC tissues (Fig. 5G). Subsequently, to further investigate whether matrix stiffness involves in the regulation of STEAP3, we observed its expression in specimens of cirrhotic human HCC (patient number = 3). Representative picture of HE staining displayed obvious tumor characteristics in cirrhotic HCC specimen (Additional file 5: Fig. S4B). Compared with the TC region, immunofluorescent staining images showed that STEAP3 was decreased in the IF region of HCC tissues (Fig. 5H, I), indicating that matrix stiffness predisposes the expression of STEAP3.

### Matrix stiffness regulates ferroptosis in HCC cells via STEAP3

**Fig. 6:**
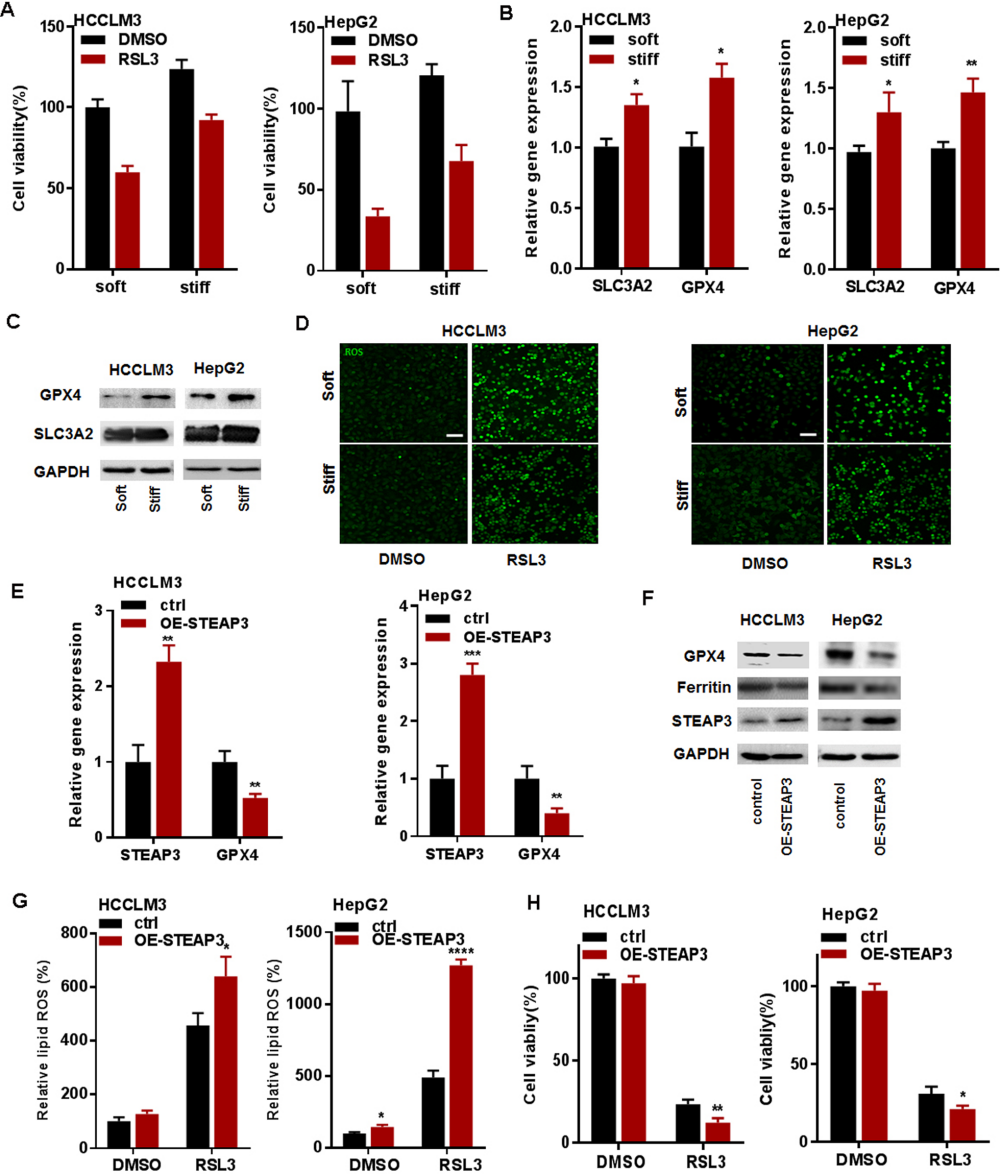
Matrix stiffness regulates ferroptosis in HCC cells via STEAP3. **A** CCK-8 assay results showing the effect of matrix stiffness on RSL3-induced cell viability in HCCLM3 and HepG2 cells, respectively. Indicated cells were treated with RSL3 (10 μM and 1 μM) for 24 h. **B**, **C** The mRNA and protein expression level of SLC3A2 and GPX4 in HCCLM3 and HepG2 cells cultured on different stiff PVA gels (2 and 40 kPa). **D** The level of RSL3-induced lipid ROS in HCCLM3 and HepG2 cells cultured on the different stiff PVA gels (2 and 40 kPa). Indicated HCCLM3 and HepG2 cells were treated with 10 μM and 1 μM RSL3 for 24 h. **E**, **F** The mRNA and protein expression level of GPX4 and ferritin in STEAP3-overexpressed HCCLM3 and HepG2 cells on a stiff matrix gel (40 kPa). **G** The level of lipid ROS in STEAP3-overexpressed HCCLM3 and HepG2 cells on a stiff matrix gel (40 kPa). **H** CCK-8 assay results showing the effect of overexpression of STEAP3 on RSL3-induced cell viability in HCCLM3 and HepG2 cells, respectively. All data were presented as mean ± SEM, N ≥ 3. *p < 0.05; **p < 0.01; ***p < 0.001; ****p < 0.0001

To investigate ferroptosis of HCC cells, HCCLM3 and HepG2 cells were treated by 0, 1, 5, and 10 μM RSL3 (ferroptosis activators) for 24 h. The CCK-8 assay results showed that cell viability was significantly decreased with the increased concentration of RSL3 in HCCLM3 and HepG2 cells (Additional file 6: Fig. S5A, B). The mRNA level of STEAP3 was increasingly induced by RSL3 in a dose-dependent manner (Additional file 6: Fig. S5C, D), indicating that 10 µM and 1 µM RSL3 is available for the induction of ferroptosis of HCCLM3 and HepG2 cells. To verify whether matrix stiffness regulates ferroptosis of HCC cells, HCCLM3 and HepG2 cultured on the soft (2 kPa) and stiff (40 kPa) PVA hydrogel were treated by RSL3 treatment for 24 h. The CCK-8 assay results showed that stiff matrix significantly impaired RSL3-reduced cell activity in HCCLM3 and HepG2 cells (Fig. 6A). Meanwhile, stiff substrate significantly increased the mRNA and protein expression of SLC3A2 and GPX4 (Fig. 6B, C), and reduced ROS production, a ferroptosis-related downstream maker, in HCCLM3 and HepG2 cells (Fig. 6D and Additional file 7: Fig. S6A, B). These results identified matrix stiffness as the key mediator of ferroptosis in HCC cells.

To further clarify whether matrix stiffness regulates ferroptosis via STEAP3 in HCC cells, overexpression of STEAP3 in HCCLM3 and HepG2 cells were performed. The results of qRT-PCR and western blot showed that overexpression of STEAP3 significantly inhibited the expression of GPX4 and ferritin on a stiff matrix (Fig. 6E, F). To evaluate STEAP3 functions as a ferrireductase, the level of ROS production was quantified by DCFH-DA in HCCLM3 and HepG2 cells. The results showed that the level of ROS was significantly up-regulated in STEAP3-overexpressed HCCLM3 and HepG2 cells (Fig. 6G). Subsequently, the CCK-8 assay results showed that overexpression of STEAP3 significantly enhanced RSL3-reduced cell viability in HCCLM3 and HepG2 cells (Fig. 6H). Collectively, these results suggested that matrix stiffness suppressed ferroptosis in HCC cells by downregulating STEAP3.

### STEAP3 is correlated with immune infiltration in HCC

**Fig. 7:**
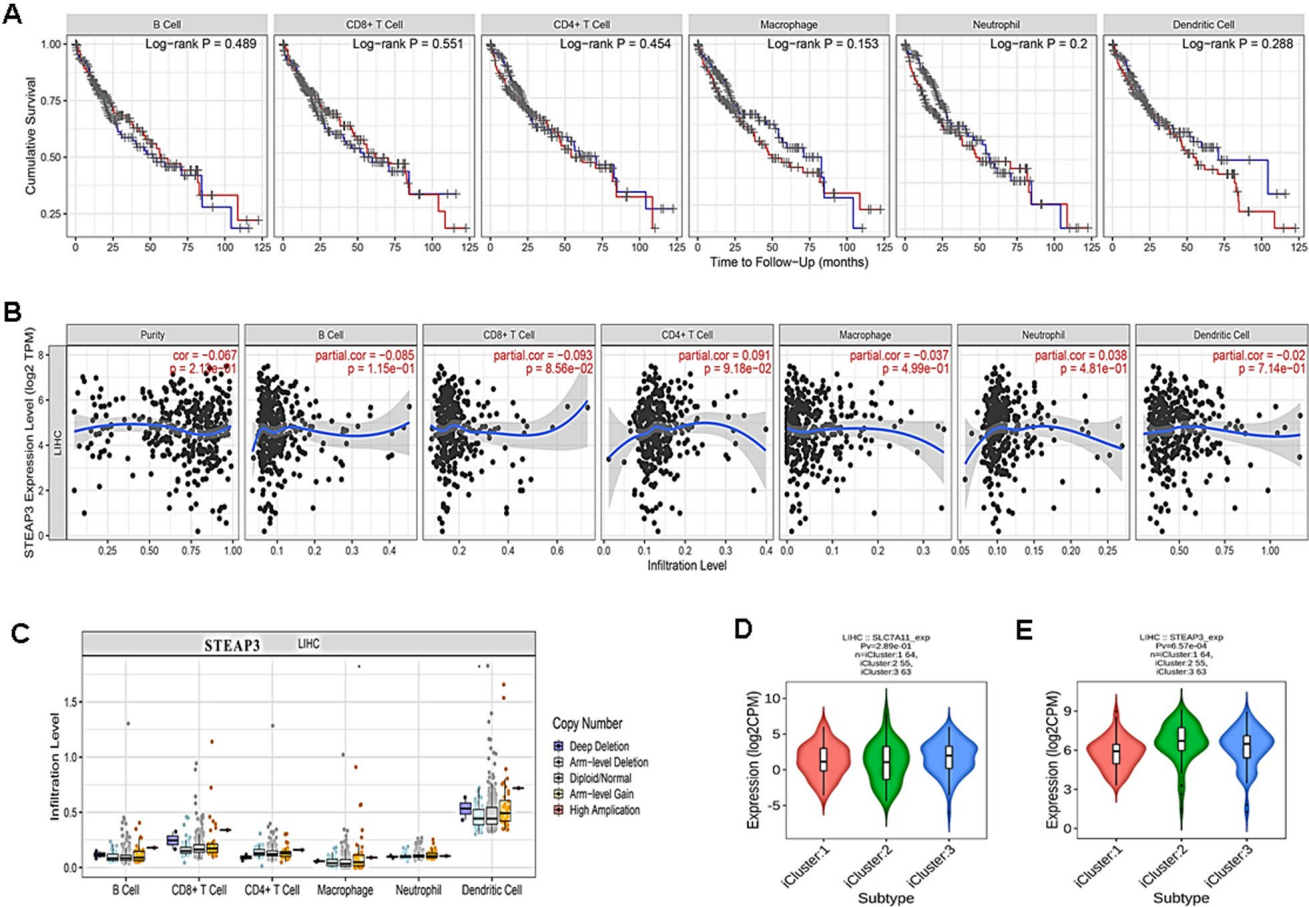
STEAP3 is correlated with immune infiltration in HCC. **A** Visual survival differences of various immune cells (B cell, CD8+ T cell, CD4+ T cell, macrophage, neutrophil, and dendritic cell) in HCC. The red line represents high level and the blue line represent low level. **B**, **C** The correlation of STEAP3 expression level vs. immune infiltration and copy number vs. immune infiltration using two-sided Wilcoxon rank sum test. **D**, **E** The correlation between STEAP3 expression level and immune subtype or molecular subtype

To further explore whether STEAP3 mediate immune infiltration, the correlation analysis was performed. The results showed that there was no significant relationship between immune infiltration of B cell, CD4+ T cell, CD8+ T cell, macrophage, dendritic cell, and neutrophil, and HCC patient survival (Fig. 7A). However, the mRNA level of STEAP3 was negatively correlated with tumor purity and the infiltration of CD8+ T cell, macrophage and dendritic cell, but positively correlated with CD4+ T cell and neutrophil (Fig. 7B). Unexpectedly, copy number of STEAP3 had no significant effect on the immune infiltration level of immune cell (Fig. 7C). Subsequently, tumor immunologically quiet, TGF-β dominant and molecular subtypes of HCC patients were analyzed. The results showed that the expression of STEAP3 was significantly correlated with immune and molecular subtypes (Fig. 7D, E). Taken together, these results demonstrated that STEAP3 directed immune microenvironment through immune infiltration in cirrhotic HCC, indicating its association with tumor immunity.

### STEAP3 mediate immunogulator PD-L2 expression in HCC

**Fig. 8:**
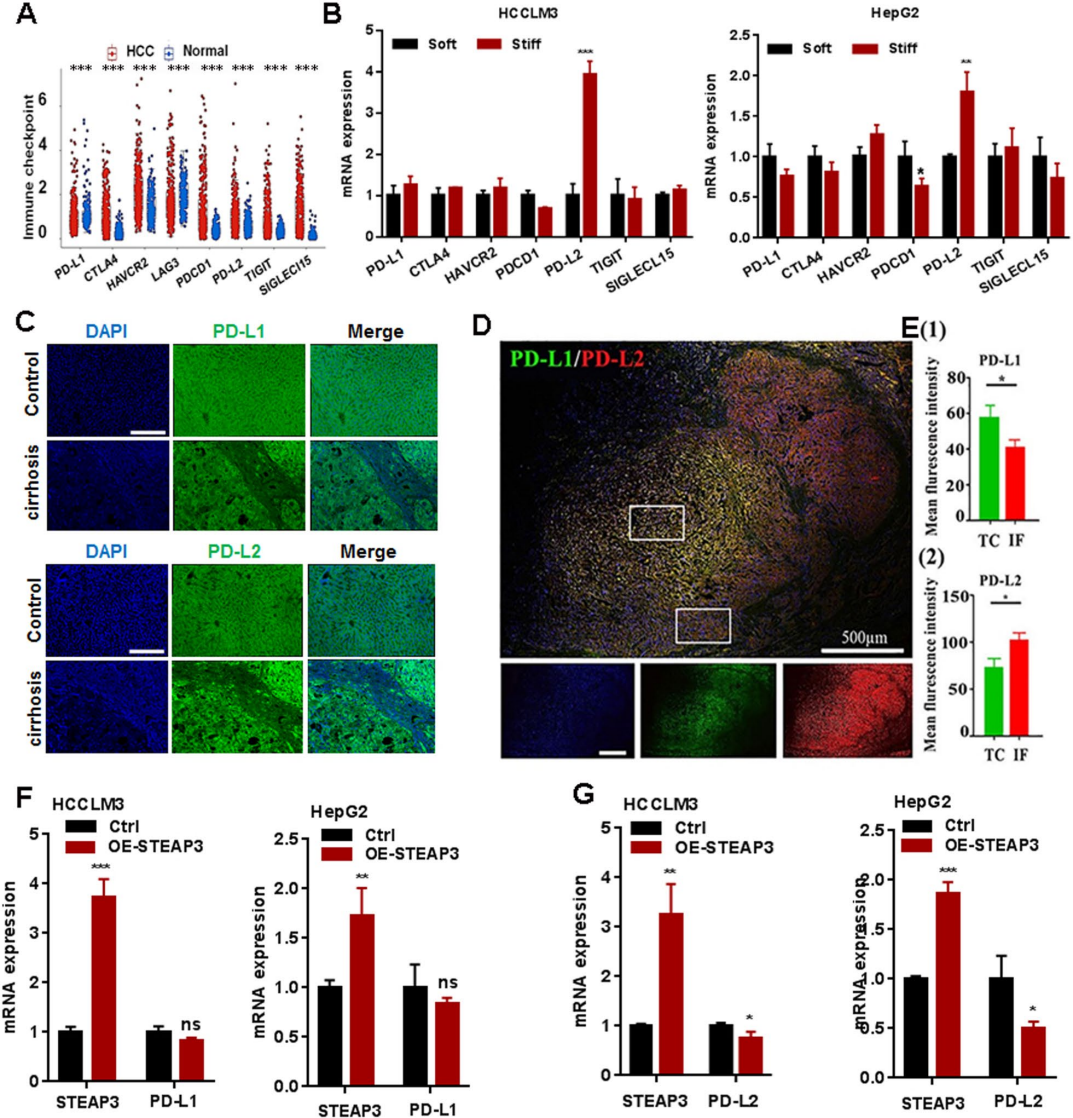
Matrix stiffness-dependent STEAP3 mediate immunogulator PD-L2 expression in HCC. **A** The expression profiles of differential immune checkpoint genes in HCC and normal liver tissues. **B** The mRNA expression level of PD-L2, CTLA4, HAVCR2, PDCD1, TIGIT, Sigleci-15 in HCCLM3 and HepG2 cells on different stiff PVA gels (2 and 40 kPa). **C** Representative immunofluorescence images showing the expression level of PD-L1 and PD-L2 in normal liver tissue and cirrhosis tissue of SD rat. **D** Representative immunofluorescence images showing the expression of PD-L1 and PD-L2 in TC and IF region of cirrhotic HCC specimens. **E** Fluorescence statistical results showing the expression level of PD-L1 and PD-L2 in IF and TC region. **F**, **G** The mRNA expression level of PD-L1 and PD-L2 in STEAP3-overexpressed HCCLM3 and HepG2 cells on a stiff matrix gel (40 kPa). All data were presented as mean ± SEM, N ≥ 3. *p < 0.05; **p < 0.01

Previous studies have proved immune checkpoints as a therapeutic target in malignancies [30]. To investigate whether STEAP3 direct tumor immunity, gene expression profile analysis of all immune checkpoints in HCC patients was firstly checked. Compared with the normal liver tissues, there was a significantly increased expression of PD-L2 (PDCD1LG2), CTLA4, HAVCR2, PDCD1, TIGIT and Siglec-15 in HCC patients, (Fig. 8A). Subsequently, the analysis results of q-PCR showed that stiff substrate significantly increased the gene expression of PD-L2 in HCCLM3 and HepG2 cells in vitro (Fig. 8B). To further evaluate the relation between matrix stiffness and PD-L2, a liver cirrhotic rat model and human cirrhotic HCC specimens were used (n = 3). Accompanied with the normal liver tissues and TC region, PD-L2 expression was significantly increased in cirrhotic tissues (Fig. 8C) and in the IF region of HCC tissues, respectively (Fig. 8D, E). Unexpectedly, there was no significantly differences for PD-L1 (CD274) in cirrhosis tissues compared with normal liver tissue. However, accompanied with the TC region, PD-L1 expression was significantly decreased in the IF region of HCC tissues. To further identify its correlation with STEAP3, the analysis of correlation coefficients was performed. The results showed that PD-L1 and PD-L2 were significant correlation with STEAP3 (Additional file 8: Fig. S7A, B). Overexpression of STEAP3 significantly suppressed the expression of PD-L2 in HCCLM3 and HepG2 cells cultured on a stiff matrix (Fig. 8G). Similarly, there were no significant differences for PD-L1 (Fig. 8F). Generally, these results suggested STEAP3 could affect tumor immune microenvironment via regulating matrix stiffness-dependent immune checkpoint receptor PD-L2 in HCC cells.

### STEAP3 supplemented with PD-L2 predict clinical benefit of sorafenib treatment in HCC patients

**Fig. 9:**
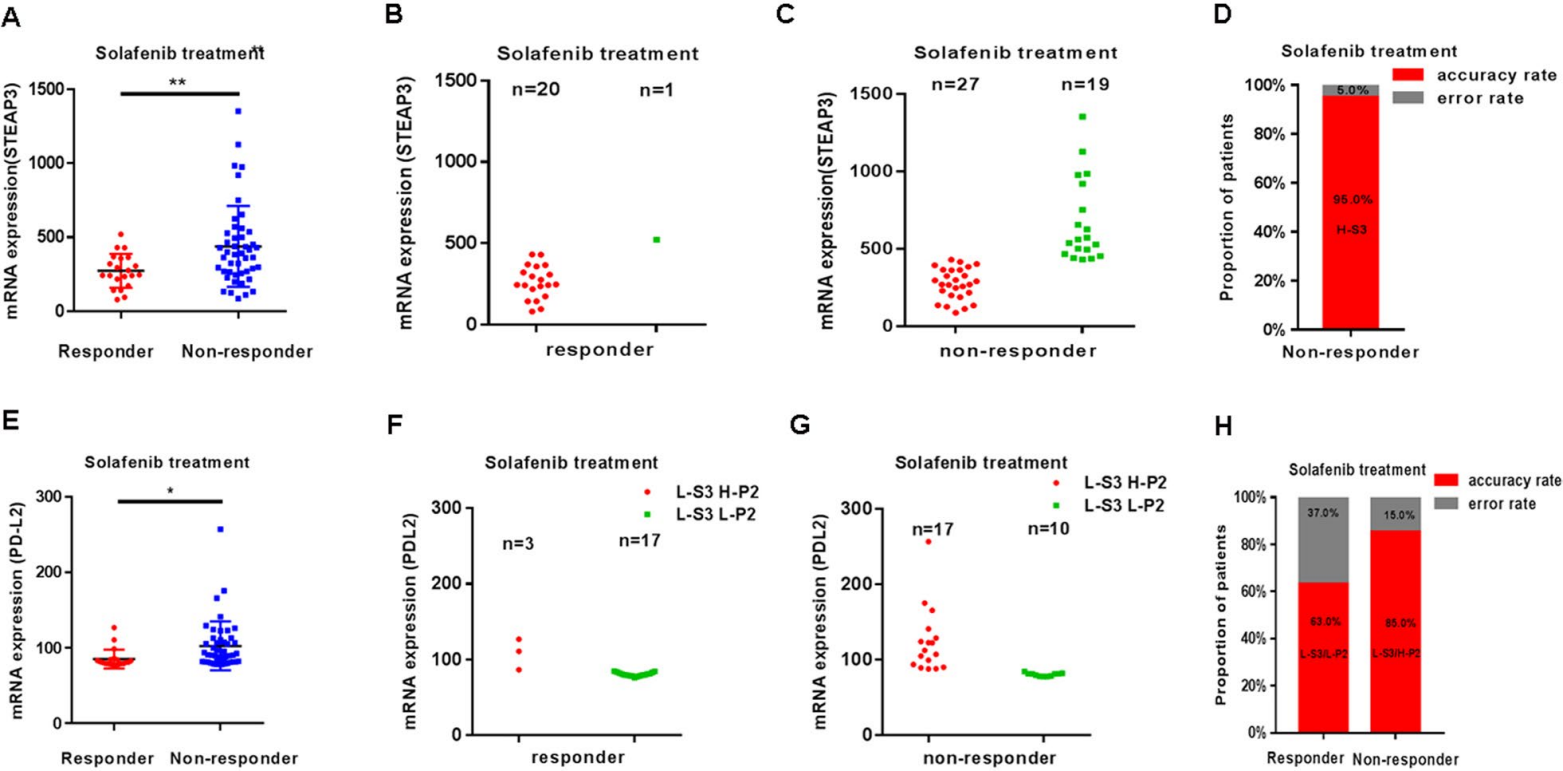
STEAP3 supplemented with PD-L2 predict sorafenib efficacy in HCC patients. **A** The differential of the gene expression of STEAP3 in sorafinib response and non-response patients. **B**, **C** The distribution of number of responders and non-responders with low/high expression of STEAP3, which is below/above the threshold. **D** The statistic results showing that the accuracy of the biomarker (STEAP3) in non-responsive HCC patients with sorafinib therapy. **E** The differential of the gene expression of PD-L2 in sorafinib response and non-response patients. **F**, **G** The distribution of number of responders and non-responders with low/high expression of PD-L2 (below/above the threshold), as STEAP3 is low expression. **L** The statistic results showing that the accuracy of these biomarkers (STEAP3 and PD-L2) in responsive and non-responsive HCC patients with sorafinib therapy

To evaluate the predictive value of STEAP3, public datasets from HCC patients with sorafenib treatment in the phase 3 STORM clinical trial was analyzed. Compared with non-responders (n = 46), the decreased levels of STEAP3 was observed in HCC responders (n = 21) following sorafenib therapy (GSE109211; Fig. 9A and Additional file 1: Table S7). To further validate it, taking the average value of STEAP3 (438.5) in non-responders as the predictive threshold, the value above the threshold was considered as high expression, and the value below the threshold considered as low expression. When it was higher than 438.5 for STEAP3, the number of responders were 1, and in non-responders were 19, respectively (Fig. 9B, C). The statistic results showed that the accuracy of these biomarkers (STEAP3) was 95.0% in non-responsive HCC patients with sorafinib therapy (Fig. 9D), indicating that high STEAP3 might be an independent biomarker of non-responder to sorafenib in HCC patients.

To improve the accuracy of prediction, based on low expression of STEAP3, matrix-dependent immune checkpoint receptor PD-L2 was used as supplementary predictive biomarker. Compared with non-responders (n = 46), the reduced expression of PD-L2 in sorafenib responders (n = 21) was observed (Fig. 9E and Additional file 1: Table S7). As above, the average value of PD-L2 (85.2) in responders was taken as the predictive threshold. When it was higher than 85.2 for PD-L2, the number of HCC sorafinib responders with in non-responders was 17 (total 20), indicating that low STEAP3/high PDL2 might also be independent biomarkers of non-responder to sorafenib in HCC patients. The number of sorafinib responders in HCC patients with low STEAP3/low PDL2 was 17 (total 27) (Fig. 9F, G), indicating that HCC patients with low PD-L2 have better clinic benefit to sorafinib. The statistic results showed that the accuracy of these biomarkers (STEAP3 and PD-L2) were 63.0% and 85.0% in responsive and non-responsive HCC patients with sorafinib therapy, respectively (Fig. 9H).

## Discussion

**Fig. 10:**
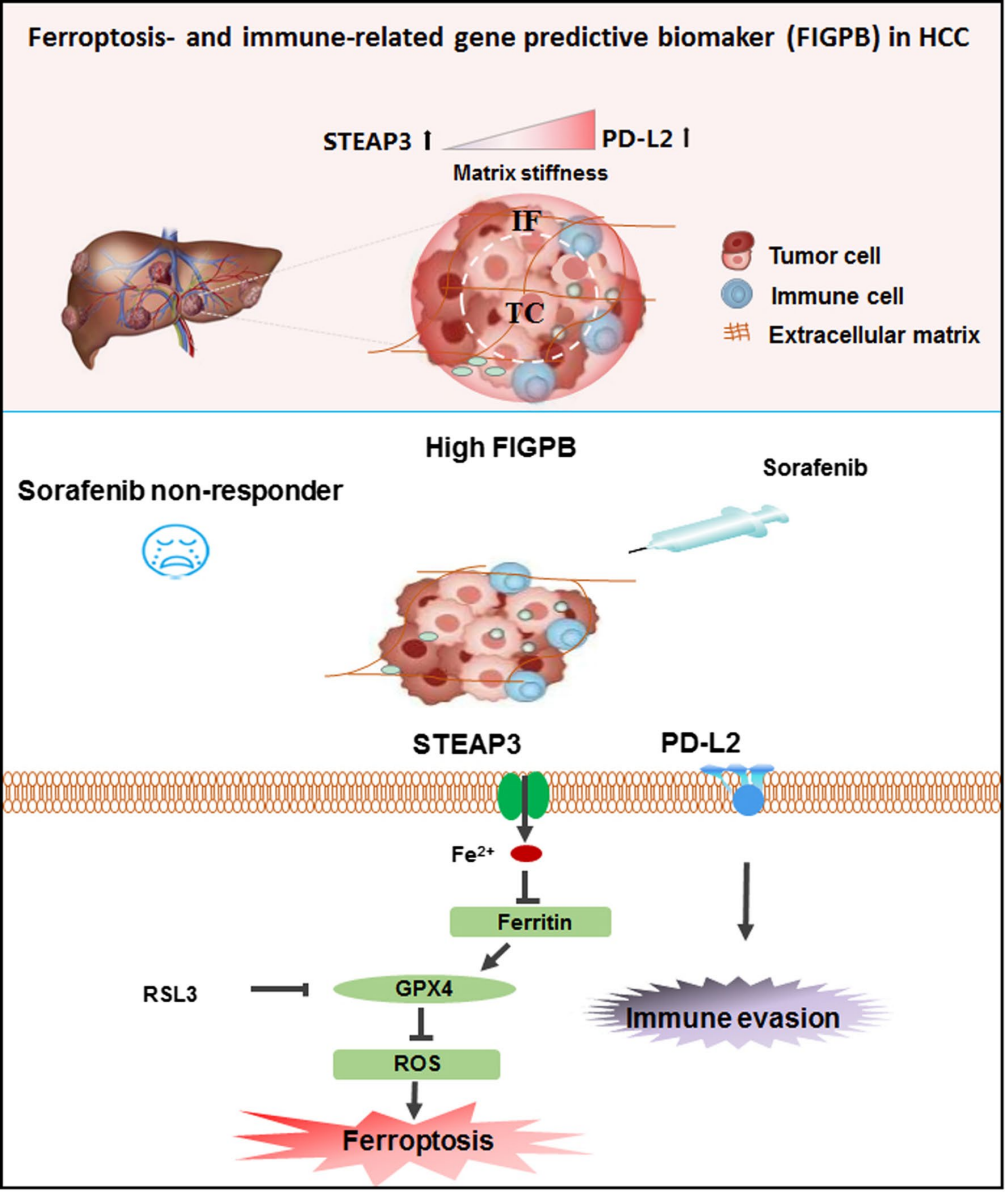
The clinical value of FIGPB in HCC patients with sorafenib therapy. The graphical abstract of the study for mechanics-driven tumor characterization of FIGPB in HCC

Previous evidences proposed that mechanical cues (e.g., matrix stiffness) of the environment orchestrates the pathogenesis and development in various cancers, including HCC, and breast cancers as well as other solid cancers [15, 31]. Ferroptosis contribute to tumorigenesis and tumor progression in HCC caused mainly liver cirrhosis, which has frequently been a potential target of chemo- and immuno-therapy [32]. However, the role of matrix stiffness on the regulation of ferroptosis and tumor immunity in HCC, and its underlying mechanism and clinical value remain elusive. Here, we identified that matrix stiffness suppressed ferroptosis of HCC cells through regulating the expression of STEAP3, which in turn shaped matrix mechanic-driven tumor immunity environment in HCC partly via PD-L2. Especially, we developed a ferroptosis- and immunity-related gene to predict a better clinical benefit to sorafenib in HCC patients (Fig. 10).

Sorafenib treatment has proven to be an effective treatment for advanced HCC patients [33]. Given that the overall response rate from sorafenib therapy is still very low [34], the identification of patients who can benefit most from these treatment is crucial. Although different prognostic or predictive biomarkers have been provided for HCC patients, a validated biomarker for predicting clinical benefits to sorafenib therapy still need to be found. Previous studies showed that multiple genes affect the development and chemotherapy resistance of tumors [35], and multivariate threshold analysis is an important approach that can help uncover candidate biomarkers or therapeutic targets [36]. In this study, based on public available ferroptosis- and immune-related gene datasets, we identified that the expression of matrix stiffness-dependent ferroptosis and immune hub genes affected HCC patients’ response to sorafenib. With the limitation of clinical cases and the lack of patient’s clinical data, such as survival time and tumor subtype, more studies remain need for clarifying.

Ferroptosis is a novel non-apoptotic form of cell death through inhibiting activating STEAP3-mediated lipid peroxidation by iron-dependent lipid reactive oxygen species (ROS) [5]. Here, we verified that matrix stiffness reduces STEAP3 expression to suppress ferroptosis of HCC cells, indicating matrix stiffness promotes HCC growth. This results are consistent with previous reports of STEAP3. STEAP3 is a major ferric reductase, which suppresses ferroptosis through deoxidizing Fe^3+^ to Fe^2+^ [37]. The reduction of STEAP3 impairs ferroptosis of tumor cells and promotes HCC progression [38]. In addition, we also demonstrated that matrix stiffness induces the expression of GPX4, a central regulator of ferroptosis-triggering mechanisms, and impairs the accumulation of ROS in HCC cells. Given the inhibition of glutathione biosynthesis and iron-dependent accumulation of lipid peroxidation kills cells undergoing ferroptosis, both glutathione biosynthesis and iron metabolism are two critical processes involved in the mechanism of ferroptosis. Therefore, our results verified that tissue mechanical cues direct ferroptosis of HCC cells in these two aspects.

Mechanical features of liver tissues play a vital role not only in tuning of physiological liver functions but also in its pathological progression. Accordingly, mechanical factors of liver tissues have been provoked as diagnostic markers and potential therapeutic targets [39, 40]. In HCC tissues, matrix stiffness from tumor central (TC) to invasion frontier (IF) area is gradually harden [41]. Consisted with this studies, our results showed an increased matrix stiffness in liver tumor tissue of DEN-induced rats. The stiffness of normal human liver tissue was shown to be approximately less than 2.5 kPa in physiological conditions, increasing to 2.5–12.5 kPa in fibrotic liver tissues, and 12.5–75 kPa in cirrhotic HCC tissues [13]. Consisted with previous studies, our results showed an increased matrix stiffness in fribrosis tissue of DEN-induced rats. Recently, polydimethylsiloxane (PDMS) substrate or other matrix gel have been designed to represent the stiffness of healthy (1–2.5 kPa) and diseased livers (10–55 kPa), respectively. Indeed, we here observed that the 40 kPa (TC) PVA substrates induces SLC7A11 expression and reduces STEAP3 expression in human HCC cells in vitro, compared with 2 kPa (IF). Similarly, we demonstrated that matrix stiffness induced the expression of immune checkpoint receptor PD-L2, not PD-L1, indicating PD-L2-dependent less anti-tumor immunity in IF area. Previous studies have demonstrated that SLC3A2 (CD98hc), ubiquitously expressed heavy subunit of heteromeric amino acid transporters that bind to an SLC7 family member (e.g., SLC7A11), was required for ECM to fully exert its structural and contributed to carcinogene [42, 43]. Overexpression of CD98hc occurs widely in HCC cells, and is associated with poor prognosis clinically [44]. Here, we observed that the expression of CD98hc was promoted by stiffer matrix. Therefore, we suspected that matrix stiffness might mediate ferroptosis to promote HCC development partly via CD98hc. In addition, many positive and negative regulators (e.g., p53, ATF3/4, Nrf2, STAT3 and BAP1) have been identified in regulating the expression and activity of SLC7A11 and STEAP3 [45–48].

Additionally, cancer immuno-therapy and prediction based on immune checkpoint inhibitors (ICIs) has achieved considerable success in the clinic [49]. However, the clinical application of ICIs was significantly limited by the fact that less than one-third of patients with most types of cancer respond to these agents. As most tumors harbor innate resistance to apoptosis, the induction of ferroptosis has gradually emerged as a novel cancer treatment strategy. Previous studies reported that STEAP3 have been implicated in the regulation of immune response in various cell types [37]. Consistent with previous studies, our results showed that the gene feature (expression level and copy number) of STEAP3 was closely correlated with the numbers of tumor-infiltrated immune cells (e.g., CD8+ T cells) in HCC. Overexpression STEAP3 impaired the expression of PD-L2 on stiffer matrix in vitro. This results indicated that STEAP3 and PD-L2 may play a synergistic role in HCC immune microenvironment. It was reported that PD-L2 expression was observed in other solid tumors, with expression detected in the absence of PD-L1 in some samples, and varied results regarding its relationship with clinical response [22]. Similarly, we demonstrated that, based on ferroptosis-related genes, PD-L2 can be used as supplementary predictive biomarker to clinical prediction of sorafenib treatment. Further understanding the landscape of tumor immune environments would be needed to help in finding new ways to treat HCC or to alter it to improve clinical benefits of chemotherapies and immunotherapies.

We and others have shown that matrix stiffness is a elegant physical environment cues constantly being challenged in physiology and disease [50]. Although the underlying mechanism remains unclear, the occurrence of ferroptosis and anti-tumor immunity inhibition associated with matrix stiffness is likely to be a common phenomenon. In normal tissues, environment factors would trigger cellular ferroptosis and adaptive immunity, contributing to developmental programs and tissue homeostasis, or even facilitating in tissue fibrosis and tumorgenesis. In contrast, in tumor tissues, in which the proliferative arrest and anti-tumor immunity has been lost, matrix stiffness-inhibited ferroptosis and immune evasion in the IF area of tumor would lead to an aberrant tumor growth, and metastasis as well as drug resistance.

## Conclusions

Our findings identify that STEAP3 characterize matrix mechanical heterogeneity and immune environment of tumor, and possess prognosis and predictive value of clinical outcome in cirrhotic HCC. Particularly, matrix stiffness impairs ferroptosis by downregulating STEAP3 in cirrhotic HCC, which in turn direct the infiltration of tumor immune cells, and exacerbate anti-tumor immunity partly via PD-L2. This finding provides a novel insights and clinical application on ferroptosis, illustrating STEAP3 supplemented with PD-L2 as the potential therapeutic targets and predictive biomarker for cirrhotic HCC. In conclusion, ferroptosis- and immune-related gene is a promising predictive biomarker for favorable sorafenib response in HCC patients.

## Supplementary Information

**Additional file 1: Table S1.** List of database. **Table S2.** Composition of the PVA hydrogel with different stiffness. **Table S3.** The primers sequences for RT-qPCR in this study. **Table S4.** Expression profile of ferroptosis-related DEGs in normal liver tissue vs. HCC from GEO datasets. **Table S5.** Expression profile of ferroptosis-related DEGs in liver cirrhosis tissue vs. HCC from GEO datasets. **Table S6.** Expression profile of ferroptosis-related DEGs in HCV-infected cirrhosis vs. HCC from GEO datasets. **Table S7.** Expression profiles of STEAP3 and PD-L2 from sorafenib-responsive and non-responsive HCC patients.

**Additional file 2: Figure S1.** Screening of the differential gene. **A**, **B** The heat map showing the differential genes between normal liver tissue and HCC (GSE45050) and between HCV-induced cirrhosis and HCC (GSE17548), respectively. Color depth represents expression.

**Additional file 3: Figure S2.** The landscape of ferroptosis-related genes in HCC. **A** Integrated plot of clinical data and ferroptosis-related genes mutation in 442 HCC samples. From top to bottom panels indicate: American joint committee on cancer tumor stage code, mutation spectrum, international classification of disease for oncology, sex, diagnosis age, overall survival, mutation count. The key to the color-coding is at the bottom. **B**, **C** The heat maps showing the mRNA expression and methylation of ferroptosis-related genes, respectively.

**Additional file 4: Figure S3.** Alteration and methylation analysis of ferroptosis-related DEGs and its correlations with survival prognosis in HCC. **A**, **B** Analysis of the mRNA expression and methylation in HCC patients with or without STEAP3 alterations. **C** Overall survival (OS) in HCC patients with or without STEAP3 alterations. **D** The heat map showing the information of 3 type’s methylations of STEAP3 in HCC. **E** The ROC curve for prediction survival prognosis of HCC.

**Additional file 5: Figure S4.** Representative HE, masson, and immunohistochemical staining images in cirrhosis and HCC tissue. **A** Representative HE staining showing tumor characteristics in cirrhotic HCC of SD rat. Normal liver tissue: the shape and size of liver cells are the same and the boundary is clear. Cirrhosis group: disordered structure of liver lobules, hyperplasia of connective tissue around veins, formation of pseudolobules (black arrows), swelling of more liver cells (blue arrows), vacuolar degeneration of a few liver cells, and round vacuoles of varying sizes (green arrows) are seen in the cytoplasm. HCC group: a large mass of tumor cells is seen locally, surrounded by connective tissue and squeezing surrounding hepatocytes (black arrow). The tumor cells have large nuclei with prominent nucleoli and slightly basophilic cytoplasm (red arrow). There is congestion in the sinusoids (yellow arrow). **B** Representative HE staining images showing tumor characteristics in cirrhotic HCC specimen. A large mass of tumor cells is seen locally, surrounded by connective tissue and squeezing surrounding hepatocytes (black arrow). The tumor cells have large nuclei with prominent nucleoli and slightly basophilic cytoplasm (red arrow).

**Additional file 6: Figure S5.** Analysis of cell viability and expression of STEAP3 in HCC cells by RSL3 treatment. **A**, **B** Cell viability of HCCLM3 and HepG2 cells with RSL3 (1–10 μM) treatment for 24 h. **C**, **D** The mRNA level of STEAP3 in HCCLM3 and HepG2 cells with RSL3 (1–10 μM) treatment for 24 h.

**Additional file 7: Figure S6.** Statistical results showing the quantitative difference in ROS level from soft and stiff HCC cells by RSL3 treatment. **A**, **B** Statistical analysis of RSL3-induced lipid ROS in HCCLM3 and HepG2 cells cultured on the different stiff PVA gels (2 and 40 kPa).

**Additional file 8: Figure S7.** Relation analysis between STEAP3 and immunomodulators. **A**, **B** Correlation analysis of STEAP3 and PD-L1 or PD-L2 in HCC.

## Data Availability

All data associated with this study are present in the paper or the Additional files.

## Abbreviations

HCC: Hepatocellular carcinoma
LC: Liver cirrhotic
NC: Normal group
HBV: Hepatitis B virus
HCV: Hepatitis C virus
DEGs: Differentially expressed genes
GEO: Gene Expression Omnibus
ECM: Extracellular matrix
TS: Tumor stages
TG: Tumor grades
OS: Overall survival
TCGA: The Cancer Genome Atlas
PVA: Polyvinylalcohol
DEN: Diethylnitrosamine
NMORN: Nitrosomorpholine
IHC: Immunohistochemical
ROC: Receiver operating characteristic
AUC: Area under the curve
GPX4: Glutathione peroxidase 4
SLC3A2: Solute carrier family 3 member 2
FIGPB: Ferroptosis- and immune-related gene predictive biomarker
