# Supplementary material for: Matrix stiffness-dependent STEAP3 coordinated with PD-L2 identify tumor responding to sorafenib treatment in hepatocellular carcinoma
Source: Cancer Cell Int. 2022 Oct 13;22:318. doi: 10.1186/s12935-022-02634-7 (PMC9563531; doi:10.1186/s12935-022-02634-7)
Supplement: Supplementary file 1 — Additional file 1: Table S1. List of database. Table S2. Composition of the PVA hydrogel with different stiffness. Table S3. The primers sequences for RT-qPCR in this study. Table S4. Expression profile of ferroptosis-related DEGs in normal liver tissue vs. HCC from GEO datasets. Table S5. Expression profile of ferroptosis-related DEGs in liver cirrhosis tissue vs. HCC from GEO datasets. Table S6. Expression profile of ferroptosis-related DEGs in HCV-infected cirrhosis vs. HCC from GEO datasets. Table S7. Expression profiles of STEAP3 and PD-L2 from sorafenib-responsive and non-responsive HCC patients. [file 12935_2022_2634_MOESM1_ESM.docx]

**Additional file 1**

**Matrix stiffness-dependent STEAP3 coordinated with PD-L2 identify tumor responding to sorafenib treatment in hepatocellular carcinoma**

Wang Shunxi^1, 2#^, Chen Long^1#^, Liu Wanqian ^1, 2,*^

^1^Key Laboratory of Biorheological Science and Technology, Ministry of Education, Bioengineering College, Chongqing University, Chongqing 400044, China.

^2^The 111 Project Laboratory of Biomechanics and Tissue Repair, College of Bioengineering, Chongqing University, Chongqing 400044, China.

^#^ Equal contribution

**Short running title:**
FIGPB as a ferroptosis- and immune-related predictive biomarker in HCC

Correspondence:

Liu Wanqian, professor

Bioengineering Institute of Chongqing University, 174 Shazheng Street, Chongqing 400000, China. <Tel:+86>15086936790. E-mail: 20201902041@cqu.edu.cn

**The Additional file contains Materials and Methods, additional tables (Table S1 to S6) and Additional figures (figures S1 to S7).**

### Cell viability assay

Cell viability was evaluated using the Cell Counting Kit-8 (CCK-8) (#C0038, Beyotime) according to the manufacturer's protocol. Simply, HepG2 and HCCLM3 cells were seeded in a 96-well plate (10,000 cells per well) and incubated at 37 °C for 24 h. Then, the cells were starved with 100 μL fresh medium containing 2% FBS, and treated with DMSO or RSL3 for 24 h. After cultured with CCK-8 reagent at 37°C in 5% CO2 for 3 hours, the cells were measured using the Tecan Safire2 Multi-Detection Microplate Reader (Morrisville, NC) at 450 nm.

### Intracellular ROS quantification

The level of intracellular reactive oxygen species (ROS) was quantified by the change of fluorescent probe 2′, 7′-dichlorofluorescin diacetate (DCFH-DA) (Sigma, Billerica, MA). Briefly, Cells were firstly treated with pcDNA3.1 (control), STEAP3-overexpressed plasmids (OE-STEAP3), and RSL3 (HCCLM3 cells: 10μm/ HepG2 cells: 1μm) for 24 h. After washed three times with PBS, cells were incubated with 10 μM DCFH-DA for 30 min at 37 °C in dark. Then, cells were washed by PBS for three times and analyzed by the microscope (Leica, Germany).

### Masson and hematoxylin-eosin staining

Rats were euthanized and perfused by PBS and paraformaldehyde successively. Then the cirrhotic liver tissues and HCC tissues were fixed, paraffin embedded, and mounted in the microtome. Consecutive sections (5 µm thickness) were taken from the cirrhotic liver tissues and HCC tissues, and stained with masson and hematoxylin/eosin. Pictures were obtained by the microscope.

### Cell transfection

STEAP3 overexpression plasmids were constructed by cloning the cDNA of the coding regions into the pcDNA3.1 (Invitrogen) vector using NheI and XhoI.

**Additional figures legends**

**Figure S1. Screening of the differential gene. (A, B)** The heat map showing the differential genes between normal liver tissue and HCC (GSE45050) and between HCV-induced cirrhosis and HCC (GSE17548), respectively. Color depth represents expression.

**Figure S2. The landscape of ferroptosis-related genes in HCC.** **(A)** Integrated plot of clinical data and ferroptosis-related genes mutation in 442 HCC samples. From top to bottom panels indicate: American joint committee on cancer tumor stage code, mutation spectrum, international classification of discase for oncology, sex, diagnosis age, overall survival, mutation count. The key to the color-coding is at the bottom. **(B, C)** The heat maps showing the mRNA expression and methylation of ferroptosis-related genes, respectively.

**Figure S3. Alteration and methylation analysis of ferroptosis-related DEGs and its correlations with survival prognosis in HCC.** **(A, B)** Analysis of the mRNA expression and methylation in HCC patients with or without STEAP3 alterations. **(C)** Overall survival (OS) in HCC patients with or without STEAP3 alterations. **(D)** The heat map showing the information of 3 type’s methylations of STEAP3 in HCC. **(E)** The ROC curve for prediction survival prognosis of HCC.

**Figure S4. Representative HE, masson, and immunohistochemical staining images in cirrhosis and HCC tissue.** **(A)** Representative HE staining showing tumor characteristics in cirrhotic HCC of SD rat. Normal liver tissue: the shape and size of liver cells are the same and the boundary is clear. Cirrhosis group: disordered structure of liver lobules, hyperplasia of connective tissue around veins, formation of pseudolobules (black arrows), swelling of more liver cells (blue arrows), vacuolar degeneration of a few liver cells, and round vacuoles of varying sizes (green arrows) are seen in the cytoplasm. HCC grouop: a large mass of tumor cells is seen locally, surrounded by connective tissue and squeezing surrounding hepatocytes (black arrow). The tumor cells have large nuclei with prominent nucleoli and slightly basophilic cytoplasm (red arrow). There is congestion in the sinusoids (yellow arrow). **(B)** Representative HE staining images showing tumor characteristics in cirrhotic HCC specimen. A large mass of tumor cells is seen locally, surrounded by connective tissue and squeezing surrounding hepatocytes (black arrow). The tumor cells have large nuclei with prominent nucleoli and slightly basophilic cytoplasm (red arrow).

**Figure S5. Analysis of cell viability and expression of STEAP3 in HCC cells by RSL3 treatment. (A, B)** Cell viability of HCCLM3 and HepG2 cells with RSL3 (1–10 μM) treatment for 24 h. **(C, D)** The mRNA level of STEAP3 in HCCLM3 and HepG2 cells with RSL3 (1–10 μM) treatment for 24 h.

**Figure S6.** **Statistical results showing the** **quantitative difference in ROS level from soft and stiff HCC cells by RSL3 treatment. (A, B)** Statistical analysis of RSL3-induced lipid ROS in HCCLM3 and HepG2 cells cultured on the different stiff PVA gels (2 and 40 kPa).

**Figure S7. Relation analysis between STEAP3 and immunomodulators. (A, B)** Correlation analysis of STEAP3 and PD-L1 or PD-L2 in HCC.

**Additional Tables**

**Table S1.** List of database.

| **Database ID** | **URL** |
| --- | --- |
| GEO dataset | https://www.ncbi.nlm.nih.gov/gds/?term= |
| TCGA | https://www.cancer.gov/ |
| cBioportal of cancer genomics | https://www.cbioportal.org/ |
| FireBrowe | http://firebrowse.org/ |
| Mexpress | https://mexpress.be/ |
| OmicShare | <http://www.omicshare.com>/ |
| TIMER | https://cistrome.shinyapps.io/timer/ |
| GEPIA | http://gepia.cancer-pku.cn/index.html/ |
| TIP | http://biocc.hrbmu.edu.cn/TIP/index.jsp/ |
| TISIDB | http://cis.hku.hk/TISIDB/index.php/ |
| Linkedomics | http://www.linkedomics.org/ |

**Table S2.** Composition of the PVA hydrogel with different stiffness.

|  | **2 kpa (soft)** | **40 kpa (stiff)** |
| --- | --- | --- |
| 8% PVA hydrogel (ml) | 10 | 10 |
| 37% HCl (ul) | 50 | 100 |
| 25% Glutaraldehyde (ul) | 32 | 160 |

**Table S3.** The primers sequences for RT-qPCR in this study.

| ***Target genes*** | ***Primer sequences*** |
| --- | --- |
| Human GAPDH | **Forward:** 5’- GGTATGACAACGAATTTGGC -3’ |
|  | **Reverse:** 5’- GAGCACAGGGTACTTTATTG -3’ |
| Human SLC7A11 | **Forward:** 5’- GGCAGTTGCTGGGCTGATTT -3’ |
|  | **Reverse:** 5’- CCATGAAGAGGCATGTGAAG -3’ |
| Human STEAP3 | **Forward:** 5’- AATGAGAGGCAGGGAGAGC -3’ |
|  | **Reverse:** 5’- CTTCAGCCAGAGGTGGGT -3’ |
| Human SLC3A2 | **Forward:** 5’- GAGGCTCCAGTCATGCTGTG -3’ |
|  | **Reverse:** 5’- TTACTCCGCTGGTCACTCAG -3’ |
| Human GPX4 | **Forward:** 5’- GCAAGGGCATCCTGGGAAA -3’ |
|  | **Reverse:** 5’- GTCCGTAGCGCTTCACCAC -3’ |
| Human PD-L1 | **Forward:** 5’- ACATGTCAGGCTGAGGGCTA -3’ |
|  | **Reverse:** 5’- TCTCTTGGAATTGGTGGTGGTG-3’ |
| Human PD-L2 | **Forward:** 5’- CAGGACCCATCCAACTTGGC -3’ |
|  | **Reverse:** 5’- CTTAGGGCTATCACTGTGGC -3’ |
| Human CTLA4  Human HAVCR2  Human LAG3  Human PDCD1  Human TIGIT  Human SIGLEC15 | **Forward:** 5’- GTCCGGGTGACAGTGCTT -3’  **Reverse:** 5’- GGAAGGTCAACTCATTCCCC -3’  **Forward:** 5’- AACCAGCCAAGGTCACCC -3’  **Reverse:** 5’- TCCCCTGGTGGTAAGCAT-3’  **Forward:** 5’- ATCACAGTGACTCCCAAATCCT-3’  **Reverse:** 5’- TTGTCCAGATACTGGAGTCACC -3’  **Forward:** 5’- TGGTGACCGAAGGGGACAAC -3’  **Reverse:** 5’- TGCGGTACCAGTTTAGCACG-3’  **Forward:** 5’- ATTCCATTGCTTGGAGCCAT -3’  **Reverse:** 5’- CTTTCTAGTCAACGCGACCA-3’  **Forward:** 5’- GGGTTCTCCCGACAGGC -3’  **Reverse:** 5’- CGAGCTGTGCACCTCTG-3’ |

**Table S4.** Expression profile of ferroptosis-related DEGs in normal liver tissue vs. HCC from GEO datasets.

GSE45050 sample value for SLC7A11

| Sample | Title | Value |
| --- | --- | --- |
| [GSM1096616](https://www.ncbi.nlm.nih.gov/geo/query/acc.cgi?acc=GSM1096616) | non-tumor liver CR562704 | 4.91784 |
| [GSM1096617](https://www.ncbi.nlm.nih.gov/geo/query/acc.cgi?acc=GSM1096617) | non-tumor liver CR562969 | 4.64136 |
| [GSM1096619](https://www.ncbi.nlm.nih.gov/geo/query/acc.cgi?acc=GSM1096619) | non-tumor liver CR561944 | 4.90035 |
| [GSM1096615](https://www.ncbi.nlm.nih.gov/geo/query/acc.cgi?acc=GSM1096615) | hepatocellular carcinoma CR560983 | 10.6826 |
| [GSM1096618](https://www.ncbi.nlm.nih.gov/geo/query/acc.cgi?acc=GSM1096618) | hepatocellular carcinoma CR562705 | 7.23561 |
| [GSM1096621](https://www.ncbi.nlm.nih.gov/geo/query/acc.cgi?acc=GSM1096621) | hepatocellular carcinoma CR561021 | 9.30179 |
| [GSM1096622](https://www.ncbi.nlm.nih.gov/geo/query/acc.cgi?acc=GSM1096622) | hepatocellular carcinoma CR559270 | 8.522 |
| [GSM1096624](https://www.ncbi.nlm.nih.gov/geo/query/acc.cgi?acc=GSM1096624) | hepatocellular carcinoma CR562998 | 6.63323 |
| [GSM1096629](https://www.ncbi.nlm.nih.gov/geo/query/acc.cgi?acc=GSM1096629) | hepatocellular carcinoma CR562996 | 8.78554 |

GSE45050 sample value for STEAP3

| Sample | Title | Value |
| --- | --- | --- |
| [GSM1096616](https://www.ncbi.nlm.nih.gov/geo/query/acc.cgi?acc=GSM1096616) | non-tumor liver CR562704 | 10.3935 |
| [GSM1096617](https://www.ncbi.nlm.nih.gov/geo/query/acc.cgi?acc=GSM1096617) | non-tumor liver CR562969 | 10.2948 |
| [GSM1096619](https://www.ncbi.nlm.nih.gov/geo/query/acc.cgi?acc=GSM1096619) | non-tumor liver CR561944 | 10.1782 |
| [GSM1096615](https://www.ncbi.nlm.nih.gov/geo/query/acc.cgi?acc=GSM1096615) | hepatocellular carcinoma CR560983 | 8.37179 |
| [GSM1096618](https://www.ncbi.nlm.nih.gov/geo/query/acc.cgi?acc=GSM1096618) | hepatocellular carcinoma CR562705 | 9.45803 |
| [GSM1096621](https://www.ncbi.nlm.nih.gov/geo/query/acc.cgi?acc=GSM1096621) | hepatocellular carcinoma CR561021 | 8.37065 |
| [GSM1096622](https://www.ncbi.nlm.nih.gov/geo/query/acc.cgi?acc=GSM1096622) | hepatocellular carcinoma CR559270 | 8.98764 |
| [GSM1096624](https://www.ncbi.nlm.nih.gov/geo/query/acc.cgi?acc=GSM1096624) | hepatocellular carcinoma CR562998 | 7.35534 |
| [GSM1096629](https://www.ncbi.nlm.nih.gov/geo/query/acc.cgi?acc=GSM1096629) | hepatocellular carcinoma CR562996 | 9.90461 |

**Table S5.** Expression profile of ferroptosis-related DEGs in liver cirrhosis tissue vs. HCC from GEO datasets.

GSE45050 sample value for SLC7A11

| Sample | Title | Value |
| --- | --- | --- |
| [GSM1096614](https://www.ncbi.nlm.nih.gov/geo/query/acc.cgi?acc=GSM1096614) | Cirrhosis of liver CR561125 | 5.18816 |
| [GSM1096620](https://www.ncbi.nlm.nih.gov/geo/query/acc.cgi?acc=GSM1096620) | Cirrhosis of liver CR561042 | 5.86519 |
| [GSM1096626](https://www.ncbi.nlm.nih.gov/geo/query/acc.cgi?acc=GSM1096626) | Cirrhosis of liver CR562008 | 4.82482 |
| [GSM1096627](https://www.ncbi.nlm.nih.gov/geo/query/acc.cgi?acc=GSM1096627) | Cirrhosis of liver CR562293 | 4.94683 |
| [GSM1096628](https://www.ncbi.nlm.nih.gov/geo/query/acc.cgi?acc=GSM1096628) | Cirrhosis of liver CR562477 | 5.79739 |
| [GSM1096615](https://www.ncbi.nlm.nih.gov/geo/query/acc.cgi?acc=GSM1096615) | hepatocellular carcinoma CR560983 | 10.6826 |
| [GSM1096618](https://www.ncbi.nlm.nih.gov/geo/query/acc.cgi?acc=GSM1096618) | hepatocellular carcinoma CR562705 | 7.23561 |
| [GSM1096621](https://www.ncbi.nlm.nih.gov/geo/query/acc.cgi?acc=GSM1096621) | hepatocellular carcinoma CR561021 | 9.30179 |
| [GSM1096622](https://www.ncbi.nlm.nih.gov/geo/query/acc.cgi?acc=GSM1096622) | hepatocellular carcinoma CR559270 | 8.522 |
| [GSM1096624](https://www.ncbi.nlm.nih.gov/geo/query/acc.cgi?acc=GSM1096624) | hepatocellular carcinoma CR562998 | 6.63323 |
| [GSM1096629](https://www.ncbi.nlm.nih.gov/geo/query/acc.cgi?acc=GSM1096629) | hepatocellular carcinoma CR562996 | 8.78554 |

GSE45050 sample value for STEAP3

| Sample | | Title | Value | |
| --- | --- | --- | --- | --- |
| [GSM1096614](https://www.ncbi.nlm.nih.gov/geo/query/acc.cgi?acc=GSM1096614) | Cirrhosis of liver CR561125 | | | 10.192 |
| [GSM1096620](https://www.ncbi.nlm.nih.gov/geo/query/acc.cgi?acc=GSM1096620) | Cirrhosis of liver CR561042 | | | 10.4819 |
| [GSM1096626](https://www.ncbi.nlm.nih.gov/geo/query/acc.cgi?acc=GSM1096626) | Cirrhosis of liver CR562008 | | | 10.4525 |
| [GSM1096627](https://www.ncbi.nlm.nih.gov/geo/query/acc.cgi?acc=GSM1096627) | Cirrhosis of liver CR562293 | | | 10.1683 |
| [GSM1096628](https://www.ncbi.nlm.nih.gov/geo/query/acc.cgi?acc=GSM1096628) | Cirrhosis of liver CR562477 | | | 10.3858 |
| [GSM1096615](https://www.ncbi.nlm.nih.gov/geo/query/acc.cgi?acc=GSM1096615) | hepatocellular carcinoma CR560983 | | | 8.37179 |
| [GSM1096618](https://www.ncbi.nlm.nih.gov/geo/query/acc.cgi?acc=GSM1096618) | hepatocellular carcinoma CR562705 | | | 9.45803 |
| [GSM1096621](https://www.ncbi.nlm.nih.gov/geo/query/acc.cgi?acc=GSM1096621) | hepatocellular carcinoma CR561021 | | | 8.37065 |
| [GSM1096622](https://www.ncbi.nlm.nih.gov/geo/query/acc.cgi?acc=GSM1096622) | hepatocellular carcinoma CR559270 | | | 8.98764 |
| [GSM1096624](https://www.ncbi.nlm.nih.gov/geo/query/acc.cgi?acc=GSM1096624) | hepatocellular carcinoma CR562998 | | | 7.35534 |
| [GSM1096629](https://www.ncbi.nlm.nih.gov/geo/query/acc.cgi?acc=GSM1096629) | hepatocellular carcinoma CR562996 | | | 9.90461 |

**Table S6.** Expression profile of ferroptosis-related DEGs in HCV-infected cirrhosis vs. HCC from GEO datasets.

GSE17548 sample value for SLC7A11

| **Sample** | **Title** | **Value** |
| --- | --- | --- |
| [GSM437460](https://www.ncbi.nlm.nih.gov/geo/query/acc.cgi?acc=GSM437460) | Ankara-3 cirrhosis sample | 7.24789 |
| [GSM437471](https://www.ncbi.nlm.nih.gov/geo/query/acc.cgi?acc=GSM437471) | Izmir-6 cirrhosis sample | 9.00333 |
| [GSM437488](https://www.ncbi.nlm.nih.gov/geo/query/acc.cgi?acc=GSM437488) | Izmir-4 cirrhosis sample | 9.22148 |
| [GSM437461](https://www.ncbi.nlm.nih.gov/geo/query/acc.cgi?acc=GSM437461) | Ankara-3 tumor sample | 6.20353 |
| [GSM437476](https://www.ncbi.nlm.nih.gov/geo/query/acc.cgi?acc=GSM437476) | Ankara-9 tumor sample | 5.22063 |
| [GSM437489](https://www.ncbi.nlm.nih.gov/geo/query/acc.cgi?acc=GSM437489) | Izmir-4 tumor sample | 7.2733 |

GSE17548 sample value for STEAP3

| **Sample** | **Title** | **Value** |
| --- | --- | --- |
| [GSM437460](https://www.ncbi.nlm.nih.gov/geo/query/acc.cgi?acc=GSM437460) | Ankara-3 cirrhosis sample | 4.44363 |
| [GSM437471](https://www.ncbi.nlm.nih.gov/geo/query/acc.cgi?acc=GSM437471) | Izmir-6 cirrhosis sample | 4.53654 |
| [GSM437488](https://www.ncbi.nlm.nih.gov/geo/query/acc.cgi?acc=GSM437488) | Izmir-4 cirrhosis sample | 4.63505 |
| [GSM437461](https://www.ncbi.nlm.nih.gov/geo/query/acc.cgi?acc=GSM437461) | Ankara-3 tumor sample | 5.35774 |
| [GSM437476](https://www.ncbi.nlm.nih.gov/geo/query/acc.cgi?acc=GSM437476) | Ankara-9 tumor sample | 4.62119 |
| [GSM437489](https://www.ncbi.nlm.nih.gov/geo/query/acc.cgi?acc=GSM437489) | Izmir-4 tumor sample | 5.33802 |

**Table S7.** Expression profiles of STEAP3 and PD-L2 from sorafenib-responsive and non-responsive HCC patients

GSE109211 sample value for STEAP3 and PD-L2 from the responding patients

| Responding patient | STEAP3 | PDL2 |
| --- | --- | --- |
| patient1 | 218.129 | 80.866 |
| patient2 | 357.745 | 79.3206 |
| patient3 | 246.869 | 77.539 |
| patient4 | 235.152 | 83.479 |
| patient5 | 241.621 | 80.9052 |
| patient6 | 275.84 | 78.218 |
| patient7 | 431.101 | 86.4783 |
| patient8 | 295.573 | 81.2731 |
| patient9 | 306.465 | 79.7935 |
| patient10 | 172.305 | 78.6663 |
| patient11 | 244.128 | 79.6608 |
| patient12 | 242.832 | 83.0936 |
| patient13 | 365.506 | 84.2909 |
| patient14 | 142.223 | 79.4348 |
| patient15 | 429.091 | 126.849 |
| patient16 | 320.099 | 84.7384 |
| patient17 | 369.598 | 82.1114 |
| patient18 | 521.34 | 98.8303 |
| patient19 | 94.3776 | 78.4122 |
| patient20 | 142.358 | 110.809 |
| patient21 | 79.7461 | 76.0516 |

GSE109211 sample value for STEAP3 and PD-L2 from the non-responding patients

| non-responding patient | STEAP3 | PDL2 |
| --- | --- | --- |
| patient1 | 1126.59 | 88.2707 |
| patient2 | 255.888 | 78.9889 |
| patient3 | 111.23 | 81.5718 |
| patient4 | 984.21 | 113.001 |
| patient5 | 439.313 | 123.16 |
| patient6 | 124.528 | 93.9293 |
| patient7 | 625.443 | 110.555 |
| patient8 | 452.449 | 92.4339 |
| patient9 | 975.333 | 106.895 |
| patient10 | 383.587 | 112.744 |
| patient11 | 537.109 | 96.9415 |
| patient12 | 435.974 | 80.0424 |
| patient13 | 750.802 | 95.5719 |
| patient14 | 464.41 | 79.0183 |
| patient15 | 495.488 | 105.565 |
| patient16 | 85.7847 | 82.5408 |
| patient17 | 429.955 | 88.3107 |
| patient18 | 246.434 | 78.1763 |
| patient19 | 363.236 | 129.191 |
| patient20 | 134.053 | 105.351 |
| patient21 | 297.357 | 88.5065 |
| patient22 | 560.095 | 106.953 |
| patient23 | 323.599 | 124.49 |
| patient24 | 430.572 | 81.9208 |
| patient25 | 289.319 | 257.169 |
| patient26 | 401.028 | 99.9264 |
| patient27 | 132.975 | 78.1604 |
| patient28 | 228.055 | 81.7171 |
| patient29 | 919.664 | 90.5046 |
| patient30 | 295.529 | 78.4166 |
| patient31 | 269.014 | 81.8097 |
| patient32 | 216.164 | 122.775 |
| patient33 | 527.516 | 81.2676 |
| patient34 | 198.617 | 175.613 |
| patient35 | 570.485 | 80.9045 |
| patient36 | 325.148 | 107.628 |
| patient37 | 1352.85 | 89.541 |
| patient38 | 265.404 | 141.275 |
| patient39 | 500.498 | 90.8778 |
| patient40 | 269.079 | 165.79 |
| patient41 | 654.126 | 126.073 |
| patient42 | 414.802 | 79.8575 |
| patient43 | 187.488 | 89.7059 |
| patient44 | 364.054 | 82.4105 |
| patient45 | 361.263 | 84.5593 |
| patient46 | 392.296 | 90.4465 |
