# Supplementary figures and images for: Matrix stiffness-dependent STEAP3 coordinated with PD-L2 identify tumor responding to sorafenib treatment in hepatocellular carcinoma
Source: Cancer Cell Int. 2022 Oct 13;22:318. doi: 10.1186/s12935-022-02634-7 (PMC9563531; doi:10.1186/s12935-022-02634-7)

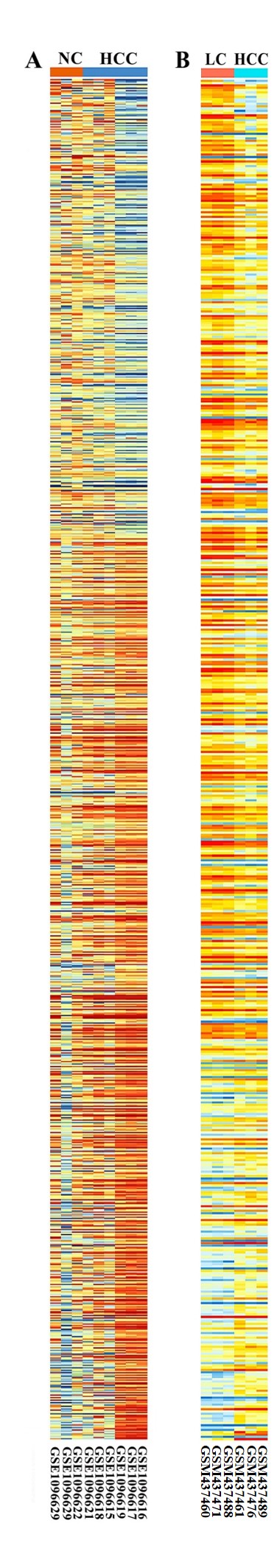

Supplement: Supplementary file 2 — Additional file 2: Figure S1. Screening of the differential gene. A, B The heat map showing the differential genes between normal liver tissue and HCC (GSE45050) and between HCV-induced cirrhosis and HCC (GSE17548), respectively. Color depth represents expression. [file 12935_2022_2634_MOESM2_ESM.jpg]

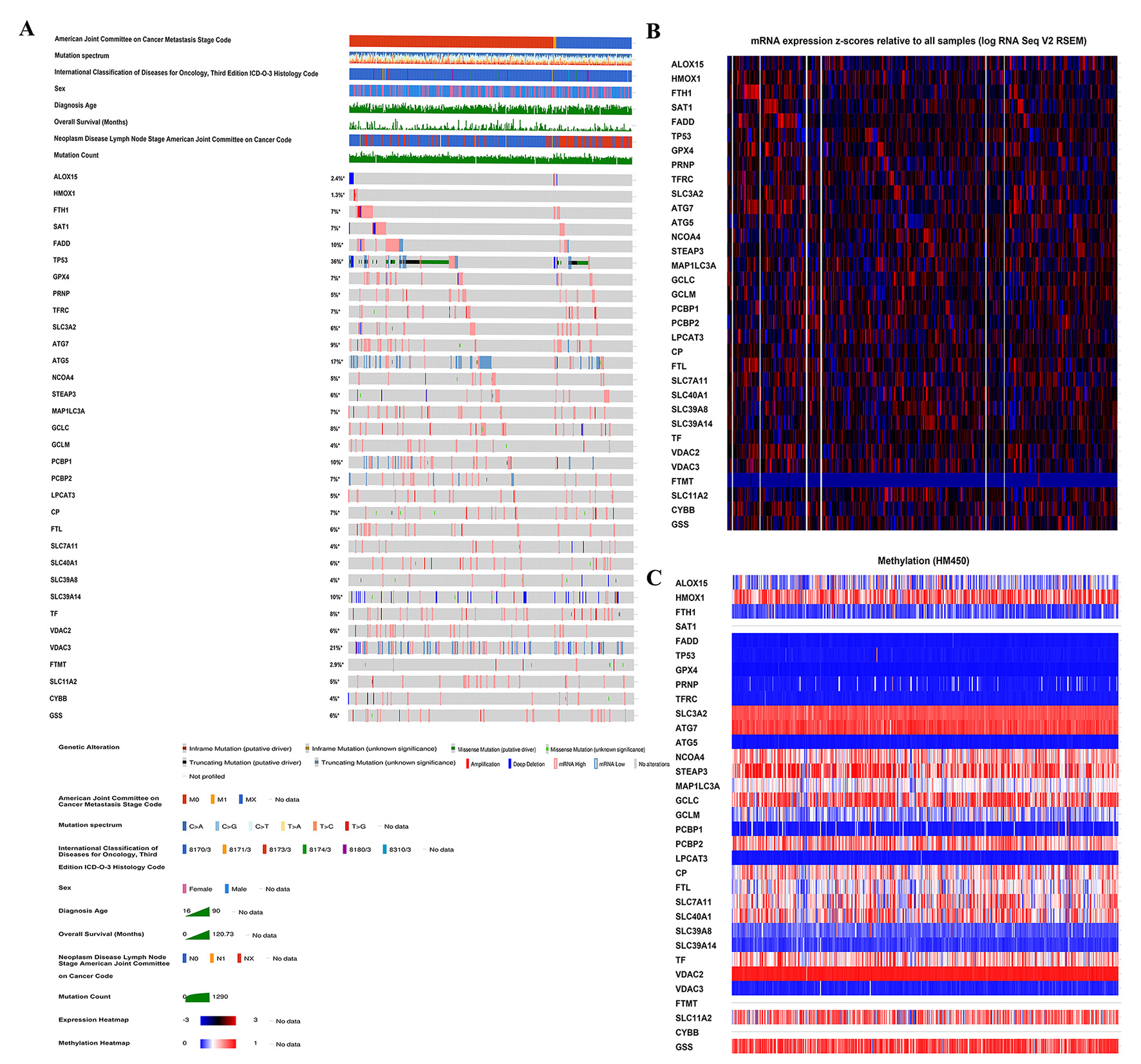

Supplement: Supplementary file 3 — Additional file 3: Figure S2. The landscape of ferroptosis-related genes in HCC. A Integrated plot of clinical data and ferroptosis-related genes mutation in 442 HCC samples. From top to bottom panels indicate: American joint committee on cancer tumor stage code, mutation spectrum, international classification of disease for oncology, sex, diagnosis age, overall survival, mutation count. The key to the color-coding is at the bottom. B, C The heat maps showing the mRNA expression and methylation of ferroptosis-related genes, respectively. [file 12935_2022_2634_MOESM3_ESM.jpg]

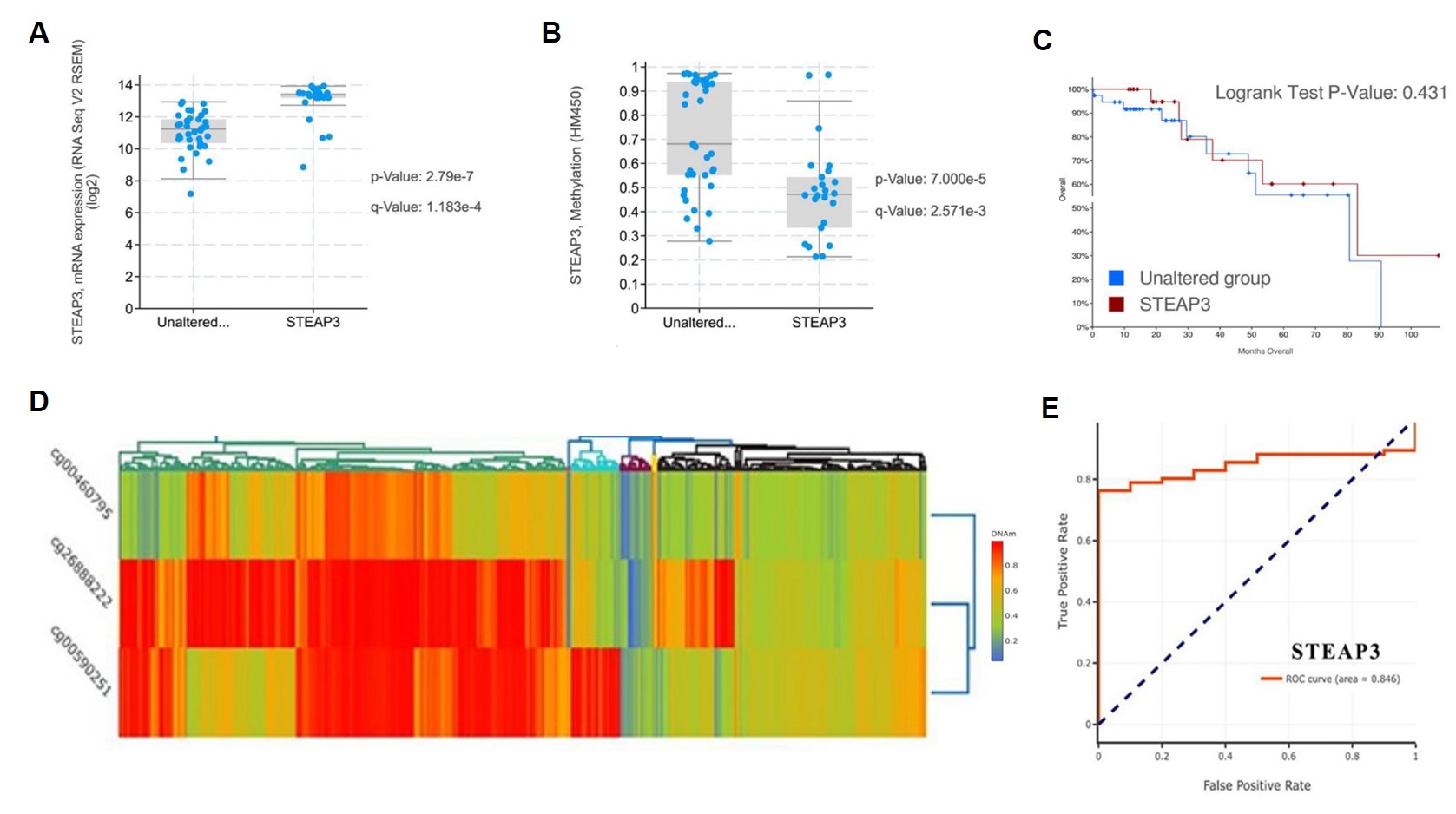

Supplement: Supplementary file 4 — Additional file 4: Figure S3. Alteration and methylation analysis of ferroptosis-related DEGs and its correlations with survival prognosis in HCC. A, B Analysis of the mRNA expression and methylation in HCC patients with or without STEAP3 alterations. C Overall survival (OS) in HCC patients with or without STEAP3 alterations. D The heat map showing the information of 3 type’s methylations of STEAP3 in HCC. E The ROC curve for prediction survival prognosis of HCC. [file 12935_2022_2634_MOESM4_ESM.jpg]

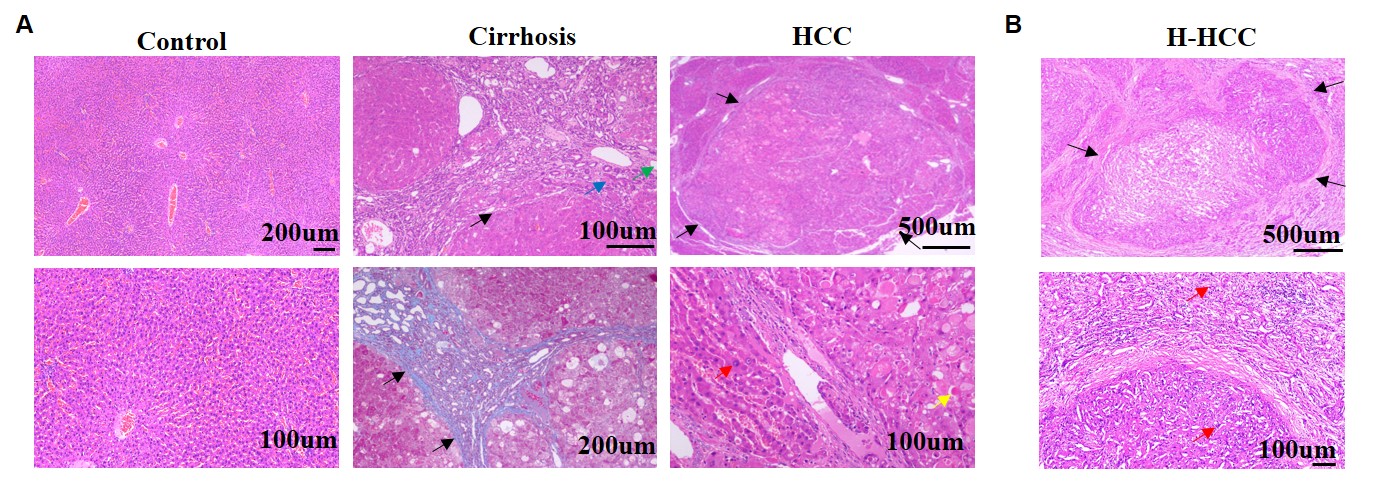

Supplement: Supplementary file 5 — Additional file 5: Figure S4. Representative HE, masson, and immunohistochemical staining images in cirrhosis and HCC tissue. A Representative HE staining showing tumor characteristics in cirrhotic HCC of SD rat. Normal liver tissue: the shape and size of liver cells are the same and the boundary is clear. Cirrhosis group: disordered structure of liver lobules, hyperplasia of connective tissue around veins, formation of pseudolobules (black arrows), swelling of more liver cells (blue arrows), vacuolar degeneration of a few liver cells, and round vacuoles of varying sizes (green arrows) are seen in the cytoplasm. HCC group: a large mass of tumor cells is seen locally, surrounded by connective tissue and squeezing surrounding hepatocytes (black arrow). The tumor cells have large nuclei with prominent nucleoli and slightly basophilic cytoplasm (red arrow). There is congestion in the sinusoids (yellow arrow). B Representative HE staining images showing tumor characteristics in cirrhotic HCC specimen. A large mass of tumor cells is seen locally, surrounded by connective tissue and squeezing surrounding hepatocytes (black arrow). The tumor cells have large nuclei with prominent nucleoli and slightly basophilic cytoplasm (red arrow). [file 12935_2022_2634_MOESM5_ESM.jpg]

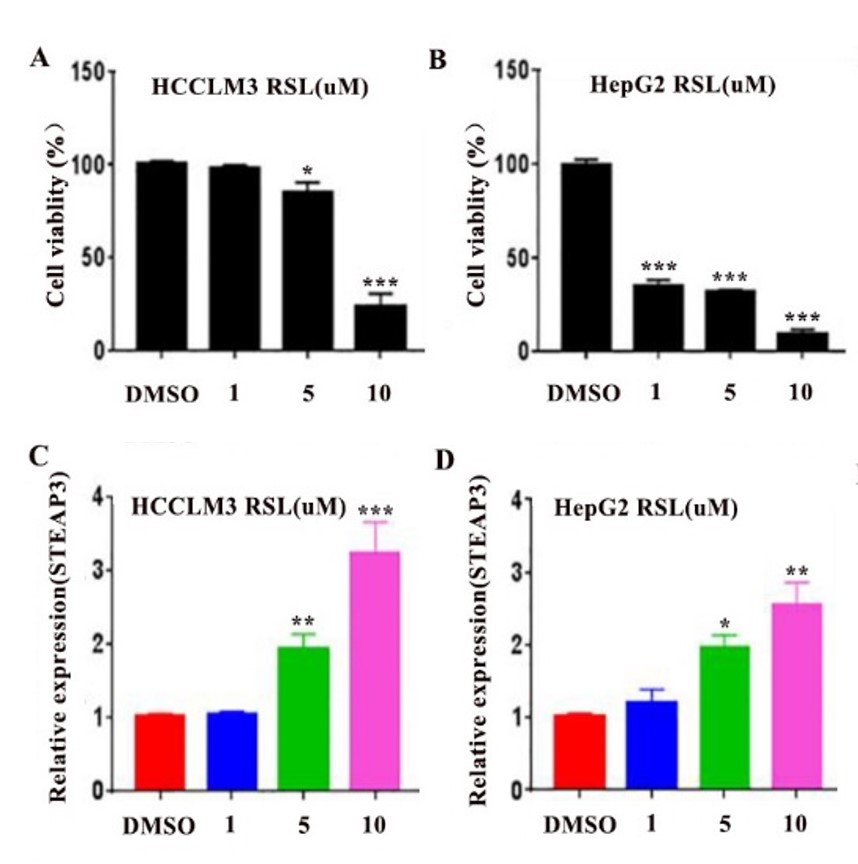

Supplement: Supplementary file 6 — Additional file 6: Figure S5. Analysis of cell viability and expression of STEAP3 in HCC cells by RSL3 treatment. A, B Cell viability of HCCLM3 and HepG2 cells with RSL3 (1–10 μM) treatment for 24 h. C, D The mRNA level of STEAP3 in HCCLM3 and HepG2 cells with RSL3 (1–10 μM) treatment for 24 h. [file 12935_2022_2634_MOESM6_ESM.jpg]

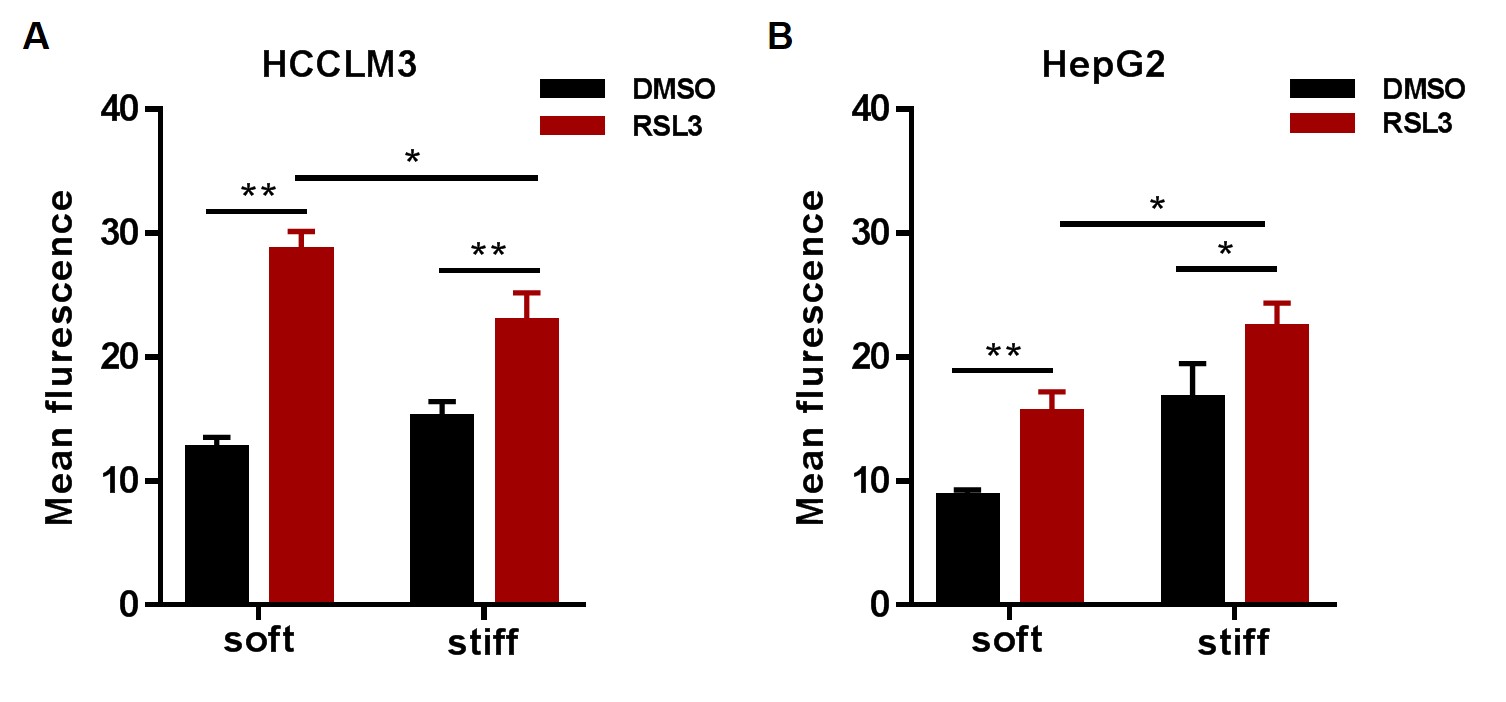

Supplement: Supplementary file 7 — Additional file 7: Figure S6. Statistical results showing the quantitative difference in ROS level from soft and stiff HCC cells by RSL3 treatment. A, B Statistical analysis of RSL3-induced lipid ROS in HCCLM3 and HepG2 cells cultured on the different stiff PVA gels (2 and 40 kPa). [file 12935_2022_2634_MOESM7_ESM.jpg]

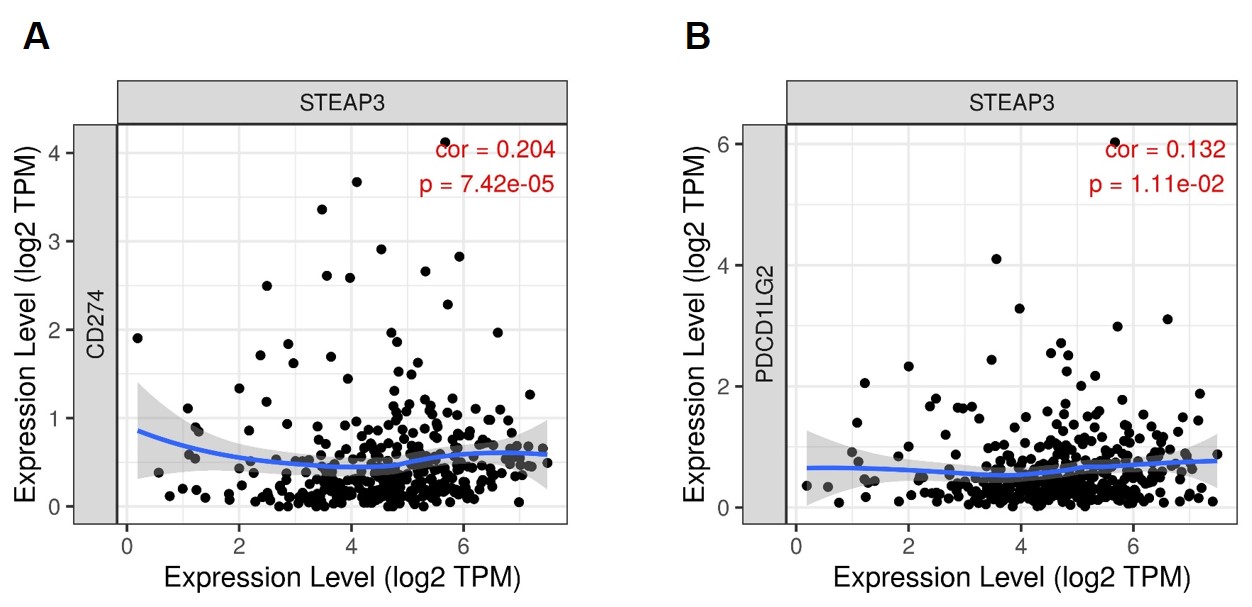

Supplement: Supplementary file 8 — Additional file 8: Figure S7. Relation analysis between STEAP3 and immunomodulators. A, B Correlation analysis of STEAP3 and PD-L1 or PD-L2 in HCC. [file 12935_2022_2634_MOESM8_ESM.jpg]
